# Supplementary material for: Midwifery care providers’ childbirth and immediate newborn care competencies: A cross-sectional study in Benin, Malawi, Tanzania and Uganda
Source: PLOS Glob Public Health. 2023 Jun 6;3(6):e0001399. doi: 10.1371/journal.pgph.0001399 (PMC10243614; doi:10.1371/journal.pgph.0001399)
Supplement: S1 Fig — (DOCX) [file pgph.0001399.s001.docx]

**S1 Fig. Skills drills summary results**

Figure 1. Skills drills assessment - clinical performance by section and mean score of tasks performed – All countries

**
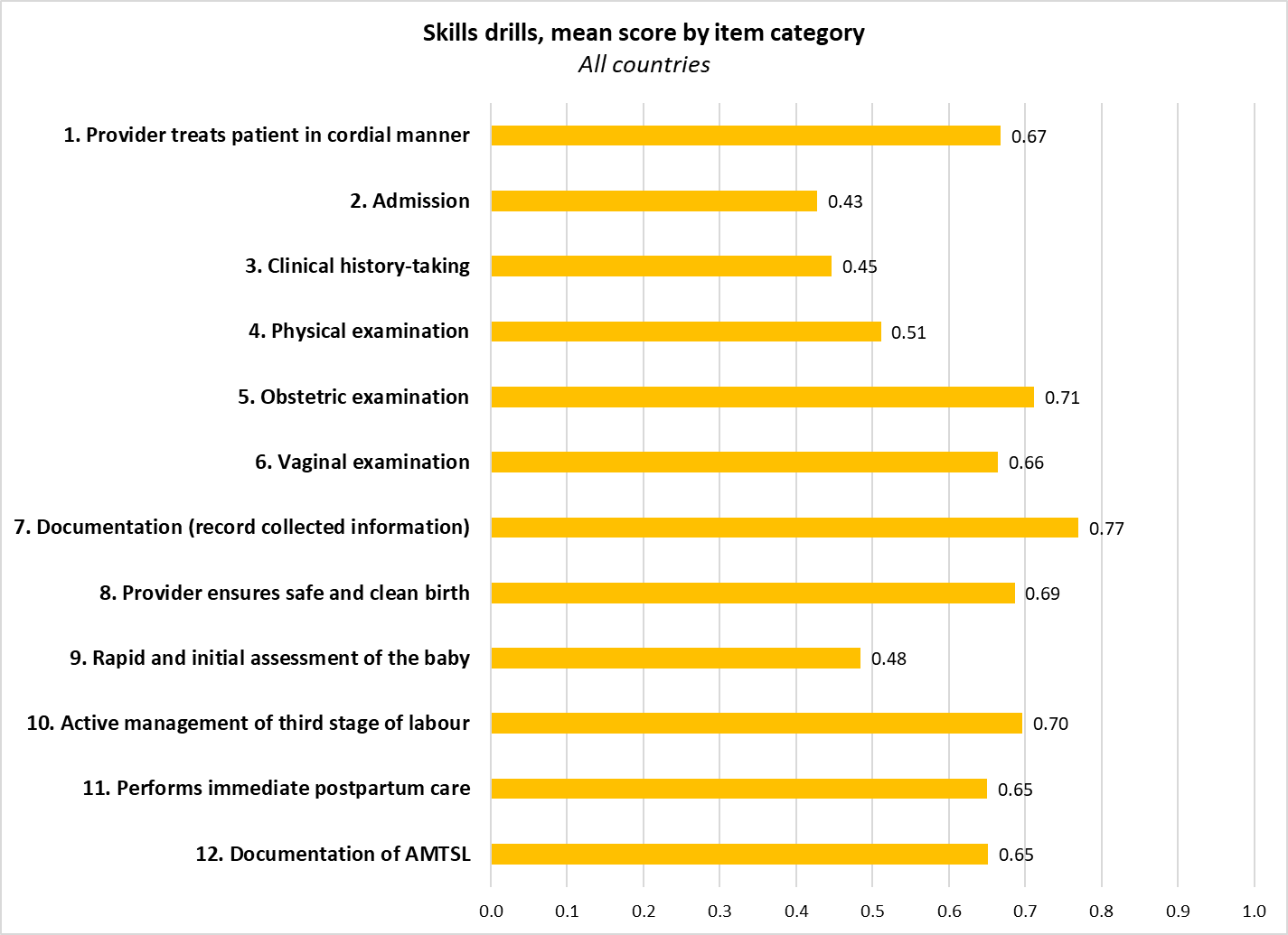
**

Figure 2. Skills drills assessment - clinical performance by section and mean score of tasks performed – Benin

**
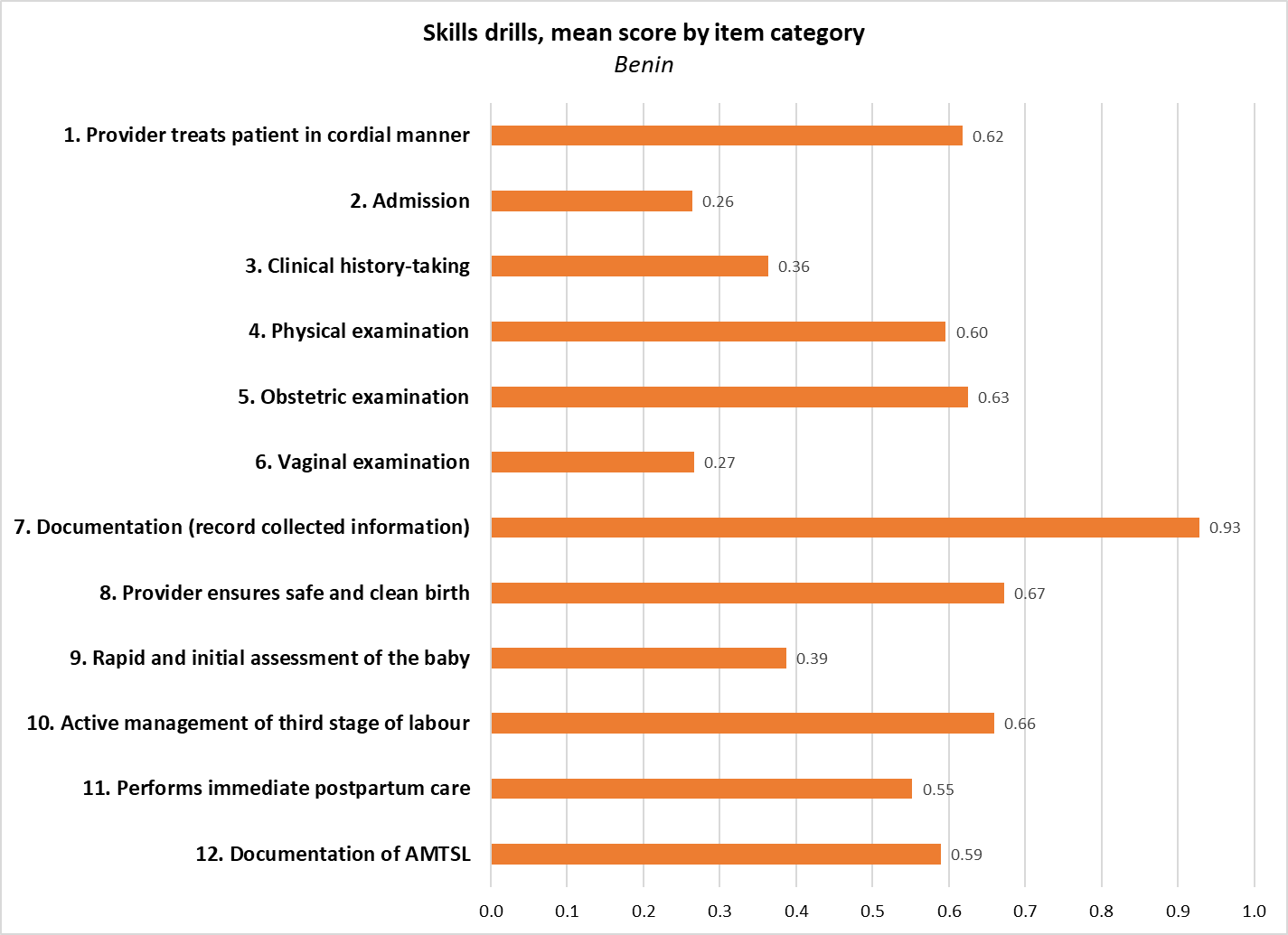
**

Figure 3. Skills drills assessment - clinical performance by section and mean score of tasks performed – Malawi

**
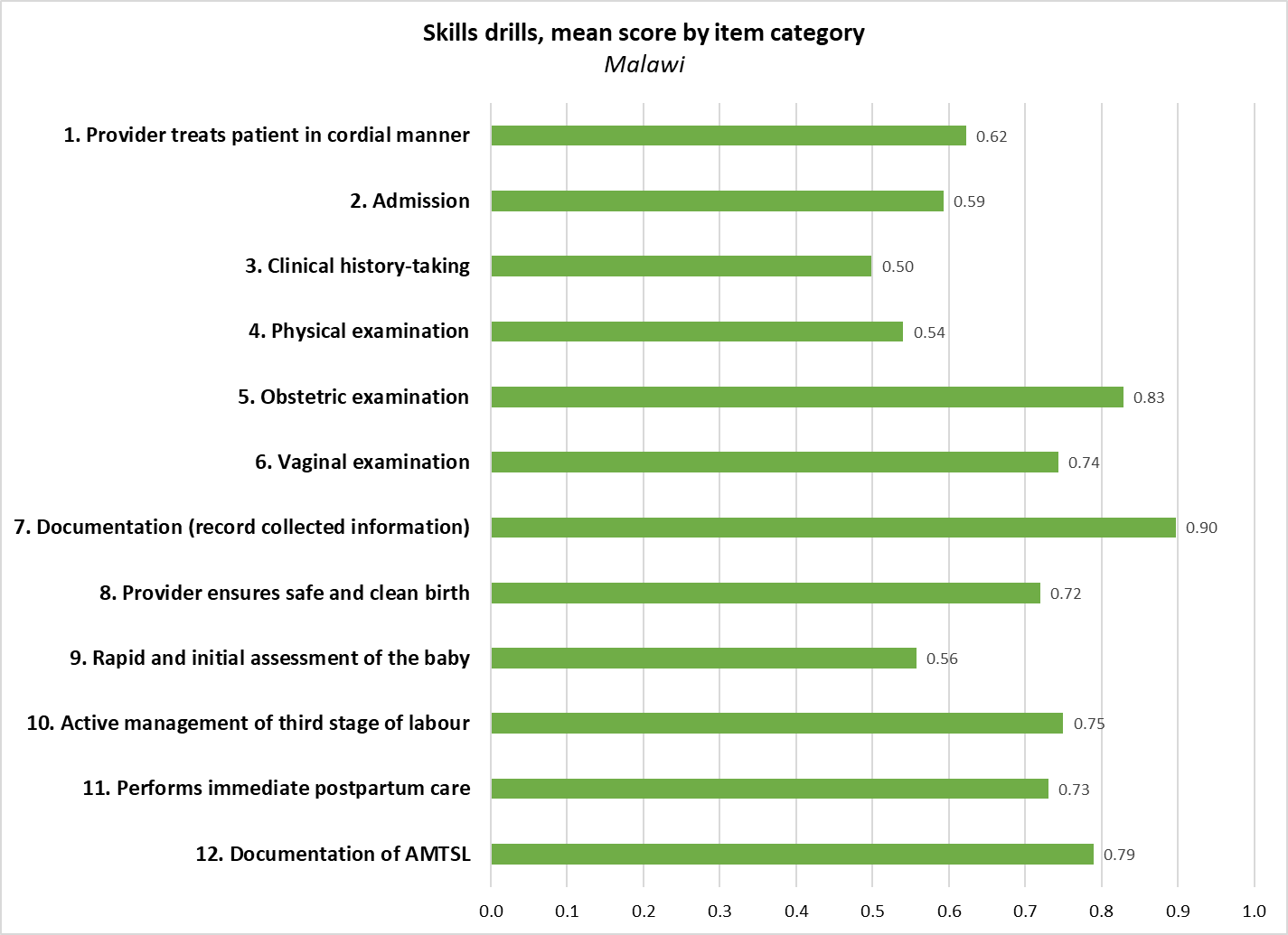
**

Figure 4. Skills drills assessment - clinical performance by section and mean score of tasks performed – Tanzania

**
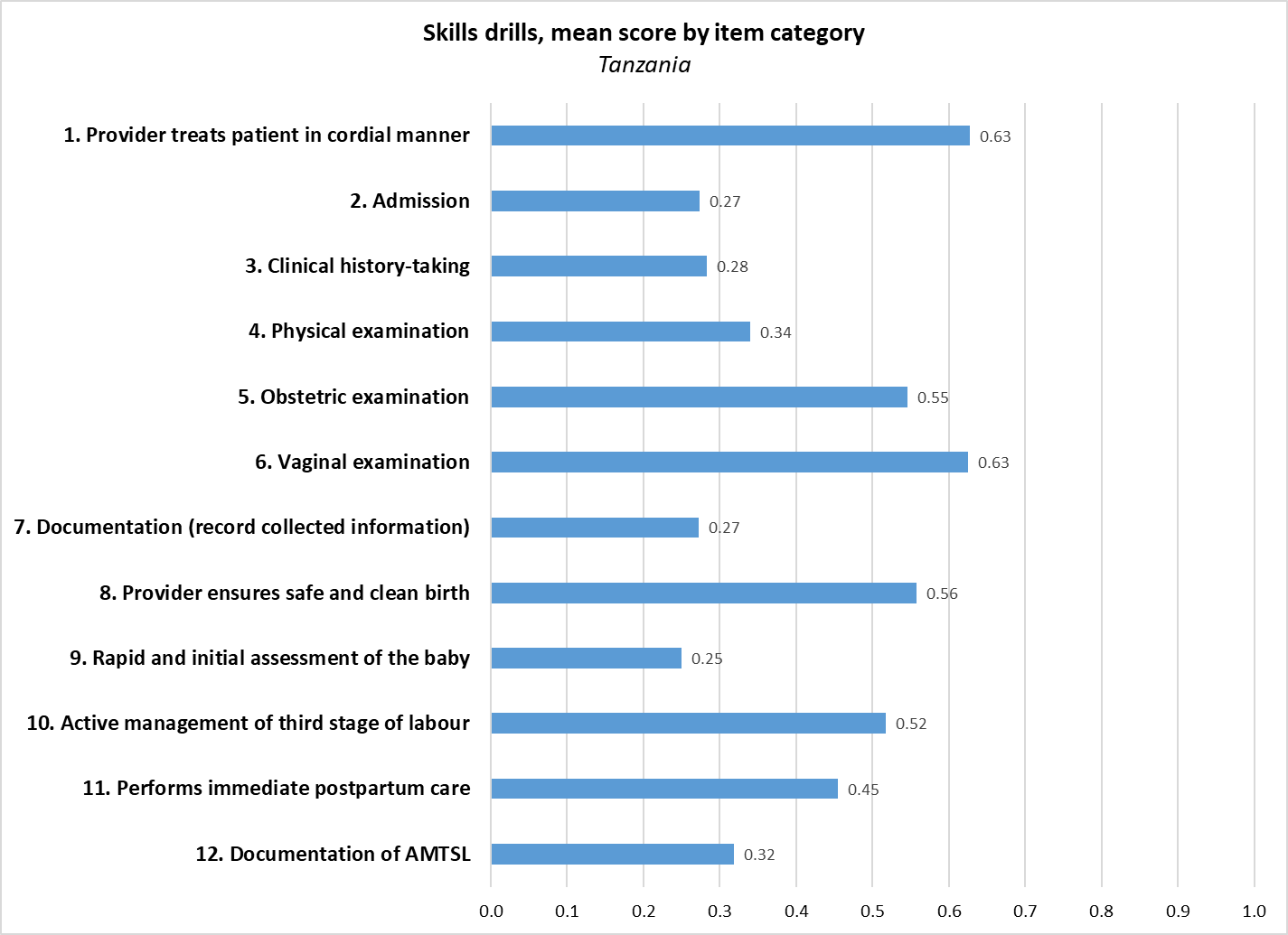
**

Figure 5. Skills drills assessment - clinical performance by section and mean score of tasks performed – Uganda

**
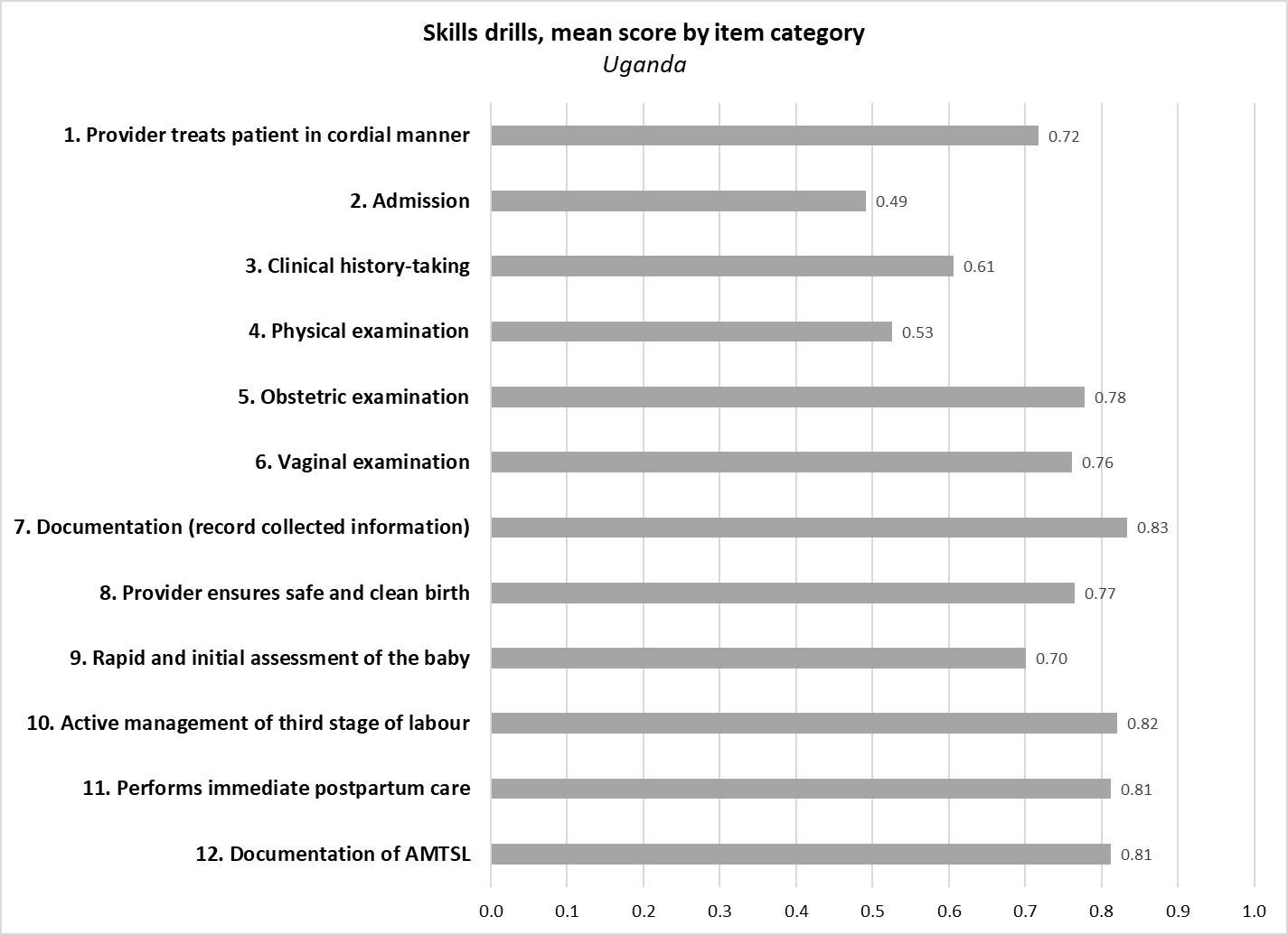
**
