# Supplementary material for: Midwifery care providers’ childbirth and immediate newborn care competencies: A cross-sectional study in Benin, Malawi, Tanzania and Uganda
Source: PLOS Glob Public Health. 2023 Jun 6;3(6):e0001399. doi: 10.1371/journal.pgph.0001399 (PMC10243614; doi:10.1371/journal.pgph.0001399)
Supplement: S2 Fig — (DOCX) [file pgph.0001399.s002.docx]

**S2 Fig. Skills drills traffic light**

Proportion of providers completing each task in the skills drills classified by level of performance – “traffic light”

| **Content of the skills drills observation checklist** | **All countries** | **Benin** | **Malawi** | **Tanzania** | **Uganda** |
| --- | --- | --- | --- | --- | --- |
|  | **Proportion of providers completing each task^a^** | | | | |
| **Provider treats patient in cordial manner (section 1)** |  |  |  |  |  |
| Greets Mary in a cordial manner | **91** | **96** | **78** | **95** | **100** |
| Introduces her/himself to Mary | **43** | **11** | **68** | **18** | **67** |
| Ensures that she/he speaks in easy-to-understand lay language (s not use technical words) with Mary | **97** | **93** | **100** | **95** | **100** |
| Explains to Mary the assessment procedures | **51** | **11** | **86** | **14** | **79** |
| Asks for Mary’s consent before performing procedures | **47** | **4** | **60** | **59** | **67** |
| Encourages Mary to ask questions | **32** | **71** | **17** | **18** | **21** |
| Listens to what the Mary has to say | **72** | **93** | **64** | **73** | **58** |
| Responds to questions and concerns | **78** | **86** | **67** | **86** | **81** |
| Provides emotional support and reassurance | **82** | **82** | **69** | **86** | **96** |
| Responds to Mary’s immediate needs (thirst, hunger, cold/hot, need to urinate, etc.) | **69** | **71** | **69** | **82** | **52** |
| **Admission (section 2)** |  |  |  |  |  |
| When assessing Mary, the provider; determines if active labour has started (fetal wellbeing, perspiration, anxiety) | **65** | **18** | **83** | **59** | **100** |
| **Asks Mary whether she has or had (section 3):** |  |  |  |  |  |
| Vaginal bleeding | **38** | **14** | **55** | **32** | **42** |
| Loss of fluid from vagina | **77** | **46** | **89** | **86** | **83** |
| Convulsions | **14** | **0** | **36** | **5** | **4** |
| Severe headache and blurred vision | **21** | **7** | **50** | **9** | **4** |
| Severe abdominal pain | **49** | **75** | **44** | **5** | **67** |
| Respiratory difficulty | **6** | **0** | **18** | **0** | **4** |
| Fever | **18** | **7** | **22** | **9** | **33** |
| Records the information on Mary’s clinical history | **66** | **64** | **84** | **24** | **79** |
| Communicates findings to Mary | **61** | **32** | **82** | **45** | **75** |
| Asks and records Mary’s clinical history following information in a non-judgemental way | **73** | **61** | **97** | **18** | **96** |
| Age of Mary | **67** | **61** | **79** | **23** | **96** |
| **Previous obstetric history:** |  |  |  |  |  |
| Number of pregnancies | **78** | **75** | **79** | **64** | **92** |
| Number of abortions | **38** | **54** | **37** | **9** | **46** |
| Number of normal births | **60** | **68** | **59** | **36** | **75** |
| Number of caesarean sections | **51** | **46** | **54** | **38** | **63** |
| Number of children born alive and still alive | **60** | **64** | **56** | **32** | **88** |
| Number of still births | **31** | **32** | **38** | **0** | **46** |
| Any maternal complication in any previous pregnancy | **44** | **21** | **54** | **32** | **67 (** |
| Date and outcome of last pregnancy | **26** | **14** | **37** | **5** | **42** |
| **Other general medical problems (for example diabetes, hypertension, asthma etc.):** |  |  |  |  |  |
| Any medication | **23** | **11** | **36** | **5** | **33** |
| Use of alternative medications or herbs | **9** | **4** | **21** | **0** | **4** |
| Receiving treatment for tuberculosis and/or HIV | **19** | **21** | **31** | **0** | **17** |
| **Clinical history-taking** |  |  |  |  |  |
| **Gestational age – ask or calculate:** |  |  |  |  |  |
| Last menstrual period (LMP) | **42** | **21** | **41** | **14** | **92** |
| Expected date of delivery (EDD) | **38** | **25** | **34** | **14** | **83** |
| **Contractions:** |  |  |  |  |  |
| Avoids asking questions during contractions | **46** | **25** | **37** | **59** | **75** |
| Asks Mary about when her labour/when the painful regular contractions began | **73** | **68** | **63** | **77** | **92** |
| Frequency and strength of the contractions | **29** | **21** | **29** | **14** | **50** |
| **Asks about Mary’s “bag of waters”/membrane:** |  |  |  |  |  |
| When the water broke? | **73** | **54** | **77** | **91** | **75** |
| What colour the water had? | **29** | **22** | **39** | **27** | **21** |
| What smell the water had? | **11 (** | **4** | **13** | **9** | **17** |
| Whether Mary feels the baby’s movements | **23** | **11** | **11** | **50** | **29** |
| Records the information on clinical history form | **63** | **68** | **76** | **14** | **79** |
| **Physical examination (section 4)** |  |  |  |  |  |
| Helps Mary onto the examination table/bed | **66** | **79** | **37** | **73** | **88** |
| Ensures that Mary remains covered with her robe or clothing | **54** | **18** | **76** | **32** | **83** |
| Asks Mary to urinate | **20** | **50** | **9** | **9** | **13** |
| Tests urine for albumin and sugar | **16** | **50** | **4** | **0** | **4** |
| Washes hands thoroughly with soap and water and dries them/uses hand gel to clean hands | **50** | **61** | **63** | **27** | **38** |
| Explains each step of the examination to Mary using easy-to-understand language | **57** | **18** | **94** | **41** | **63** |
| **Takes vital signs** |  |  |  |  |  |
| Temperature | **78** | **96** | **86** | **45** | **75** |
| Blood pressure (BP) | **92** | **96** | **89** | **91** | **92** |
| Pulse | **62** | **82** | **71** | **33** | **50** |
| Respiratory rate | **16** | **0** | **34** | **5** | **17** |
| Oedema | **34** | **71** | **26** | **9** | **25** |
| Checks the conjunctiva for anaemia | **54** | **96** | **47** | **14** | **50** |
| Observes Mary’s emotional response to labour | **65** | **57** | **57** | **64** | **88** |
| **Obstetric examination (section 5)** |  |  |  |  |  |
| Observes the shape and size of the abdomen and checks for the presence of scars | **44** | **21** | **63** | **18** | **63** |
| Determines fundal height, fetal lie and presentation, engagement of presenting part | **88** | **96** | **84** | **77** | **92** |
| Evaluates uterine contractions (frequency, strength and duration over a 10-minute period) | **41** | **25** | **70** | **14** | **38** |
| Auscultates fetal heart rate (FHR) | **96** | **96** | **100** | **95** | **92** |
| Records the results of the obstetric examination on the clinical history form | **77** | **89** | **84** | **36** | **92** |
| Communicates findings to Mary | **80** | **46** | **95** | **86** | **92** |
| **Vaginal examination (section 6)** |  |  |  |  |  |
| Puts on a clean plastic or rubber apron | **37** | **18** | **64** | **0** | **50** |
| Washes hands thoroughly with soap and water and dries them/uses hand gel to clean hands | **49** | **29** | **64** | **59** | **38** |
| Puts examination/sterile/HLD gloves on both hands | **97** | **96** | **97** | **100** | **96** |
| Asks for permission to perform a vaginal examination | **69** | **4** | **92** | **86** | **92** |
| Examines the vulva (ulcers, blood liquid, secretion, presenting part) | **42** | **32** | **51** | **27** | **50** |
| Cleanses the perineum with non-alcoholic antiseptic solution using the hand that will not be used to perform the examination | **65** | **7** | **85** | **86** | **83** |
| **Performs per vaginal examination following standard technique:** |  |  |  |  |  |
| Gently inserts two lubricated fingers of the examining hand into the vagina | **97** | **100** | **97** | **95** | **96** |
| Notes the length, texture and dilatation of the cervix | **91** | **89** | **87** | **95** | **96** |
| Notes if the membranes are intact, or if they have ruptured, ensures the cord has not prolapsed | **64** | **82** | **56** | **45** | **71** |
| Measures the level of the presenting part in centimetres above or below Mary’s ischial spines | **51** | **14** | **49** | **81** | **71** |
| Identifies fetal presentation and determines fetal position by palpating the features of the presenting part (e.g. fontanelles in cephalic presentation) | **52** | **11** | **62** | **59** | **79** |
| If gloves are disposable, places them in a waste-container with a plastic liner; OR if they are reusable, immerses them in a 0.5 chlorine solution for at least 10 minutes before transferring them for sterilization | **81** | **96** | **70** | **73** | **88** |
| Washes hands thoroughly with soap and water and dries them/uses hand gel to clean hands | **42** | **32** | **54** | **27** | **50** |
| Record the results of the vaginal examination on the clinical history form | **77** | **93** | **87** | **23** | **92** |
| Communicates findings to Mary | **81** | **46** | **100** | **82** | **92** |
| **Documentation (record collected information) (section 7)** |  |  |  |  |  |
| Record Mary’s information and assessment of findings | **77** | **93** | **90** | **27** | **83** |
| **Provider ensures safe and clean birth (section 8)** |  |  |  |  |  |
| Preparation of equipment (e.g. delivery kit, oxytocin, gloves etc.) | **89** | **86** | **92** | **77** | **100** |
| Washes hands thoroughly with soap and water and dries them/uses hand gel to clean hands | **33** | **29** | **41** | **23** | **33** |
| Puts sterile or HLD gloves on both hands | **95** | **89** | **100** | **95** | **92** |
| Puts on personal protective equipment (plastic/rubber apron) | **43** | **18** | **79** | **0** | **54** |
| Asks for permission to cleanse the perineum | **29** | **0** | **47** | **5** | **58** |
| Cleanses the perineum with water or a non-alcoholic antiseptic solution | **39** | **14** | **54** | **14** | **67** |
| Monitors FHR every five minutes during second stage | **15** | **18** | **18** | **9** | **13** |
| Supports Mary to bear down when she feels the desire in the position she chooses (does not force her to bear down) | **88** | **93** | **74** | **95** | **96** |
| Allows the head to spontaneously crown while guarding the perineum | **93** | **96** | **95** | **86** | **92** |
| After the emergence of the head, asks Mary to briefly refrain from bearing down (open mouth breathing) and explains how to perform open mouth breathing | **65** | **82** | **64** | **55** | **58** |
| Assists Mary in birthing the baby - allows spontaneous restitution and external rotation of the head without manipulation | **86** | **89** | **85** | **68** | **100** |
| Guides the baby’s head and chest in an upward curve until the posterior shoulder has emerged over the perineum | **96** | **96** | **92** | **95** | **100** |
| Holds the baby around the chest to aid the birth of the trunk and lift it towards Mary’s abdomen | **82** | **93** | **62** | **86** | **100** |
| Places the baby on a dry towel/cloth on Mary’s abdomen | **72** | **75** | **77** | **32** | **96** |
| Wipes the baby’s eyes with a clean piece of cloth | **65** | **82** | **59** | **45** | **75** |
| Waits for 1-3 minutes before clamping and cutting the cord | **74** | **96** | **71** | **45** | **79** |
| Clamps and cuts the cord using clean/sterile blade/ instrument | **97** | **100** | **95** | **95** | **100** |
| Informs Mary of the sex of her child (with same enthusiasm if male or female) | **73** | **71** | **79** | **86** | **54** |
| **Rapid and initial assessment of the baby (section 9)** |  |  |  |  |  |
| Receives the baby using a new set of gloves | **29** | **18** | **31** | **18** | **50** |
| Dries and stimulates the baby with a clean dry towel from head to feet | **77** | **71** | **89** | **45** | **96** |
| Discards the used towel and covers the baby including the head with a clean dry towel | **58** | **50** | **58** | **45** | **79** |
| Determines the APGAR score at 1 and 5 minutes | **36** | **25** | **31** | **23** | **67** |
| Encourages “Baby Crawl” practice and immediate breastfeeding | **36** | **11** | **56** | **9** | **63** |
| Notes the date and time of birth | **54** | **57** | **69** | **9** | **67** |
| **Active management of third stage of labour (section 10)** |  |  |  |  |  |
| Touches Mary’s abdomen to rule out the presence of a second baby (without stimulating contractions) | **34** | **7** | **32** | **23** | **79** |
| Give inj. Oxytocin 10 units direct I/M and wait for next contraction within one minute after birth of the baby | **98** | **100** | **95** | **100** | **100** |
| Holds the cord along with clamp and provides controlled cord traction/sustained downward traction | **96** | **96** | **97** | **86** | **100** |
| Repeats controlled cord traction while simultaneously applying counter pressure above pubis to guard uterus (places the other hand above the level of the symphysis pubis with hand facing towards the umbilicus to provide counter traction on the uterus) | **85** | **89** | **97** | **45** | **96** |
| Applies steady tension by pulling the cord firmly and maintaining pressure (jerky movements and force must be avoided) | **86** | **89** | **89** | **59** | **100** |
| When the placenta is visible at the vulva holds the placenta with both hands, assists in the expulsion of the placenta, by turning it over in the hands, without applying traction | **82** | **82** | **84** | **64** | **96** |
| Uses a gentle and upward and downward movement or twisting action to deliver the membranes, “teasing out” the membranes | **75** | **82** | **76** | **36** | **100** |
| Checks whether the uterus is well contracted | **86** | **96** | **81** | **73** | **96** |
| Massages the uterus with one hand on a cloth over the abdomen, until the uterus contracts firmly | **90** | **100** | **82** | **86** | **96** |
| Checks the placenta on a clean table or the delivery bed with a good light source: whether all the lobules are present and fit together to see if complete (missing cotyledon) and to identify any abnormalities such as aberrant vessels or nods in the umbilical cord | **68** | **57** | **73** | **59** | **79** |
| Inspects the membranes for completeness | **59** | **36** | **78** | **32** | **79** |
| Notes the position of insertion of the cord | **26** | **18** | **43** | **5** | **29** |
| Inspect the cut end of the cord for the presence of two arteries and one vein | **19** | **4** | **42** | **5** | **17** |
| **Performs immediate postpartum care (section 11)** |  |  |  |  |  |
| Ensures direct strong light into the perineum | **35** | **39** | **39** | **0** | **54** |
| Ensures Mary is in a comfortable position | **74** | **86** | **82** | **23** | **96** |
| Informs and gains consent from Mary to check her perineum | **43** | **0** | **76** | **27** | **54** |
| Inspects the lower vagina and perineum for lacerations/tear and checks for increased bleeding from the cervix/cervical tear | **88** | **67** | **95** | **91** | **96** |
| Gently cleanses the vulva and perineum with clean water or a non-alcoholic antiseptic solution and dries with a clean, soft cloth | **64** | **61** | **54** | **50** | **96** |
| Makes sure that Mary is comfortable (clean, hydrated and warmly covered) | **84** | **79** | **85** | **82** | **92** |
| **Documentation (section 12)** |  |  |  |  |  |
| Washes hands thoroughly using soap and water and dries them/uses hand gel to clean hands | **53** | **39** | **74** | **23** | **63** |
| Records relevant details (childbirth, active management of the third stage of Labour (AMTSL), placenta examination, newborn condition) on the Mary’s record | **78** | **79** | **84** | **41** | **100** |

a. Proportion of providers completing each task in the skills drills classified by level of performance: less than 50% (red) = precarious performance gap, 50% to less than 80% (yellow) = critical gap performance and higher than 80% (green) = performed in many cases to the expected performance level.
