# Supplementary material for: Midwifery care providers’ childbirth and immediate newborn care competencies: A cross-sectional study in Benin, Malawi, Tanzania and Uganda
Source: PLOS Glob Public Health. 2023 Jun 6;3(6):e0001399. doi: 10.1371/journal.pgph.0001399 (PMC10243614; doi:10.1371/journal.pgph.0001399)
Supplement: S2 Table — (DOCX) [file pgph.0001399.s004.docx]

**S2 Table. Knowledge and working environment assessment results sections 2-7**

**Results of the knowledge assessment sections 2-7. Results from section 1 are included in the main manuscript.**

| **Section 2. Working environment** | | |  |  |  |
| --- | --- | --- | --- | --- | --- |
|  | **All countries** | **Benin** | **Malawi** | **Tanzania** | **Uganda** |
|  | (n = 299) | (n= 72) | (n=98) | (n=81) | (n=48) |
| **Receiving supervision to childbirth care** |  |  |  |  |  |
| Yes | 91 (30%) | 16 (22%) | 32 (32%) | 12 (15%) | 31 (30%) |
| No | 96 (32%) | 39 (54%) | 24 (24%) | 30 (37%) | 3 (6%) |
| Sometimes | 112 (37%) | 17 (24%) | 42 (48%) | 39 (48%) | 14 (29%) |
|  |  |  |  |  |  |
| **Access to education and training resources** |  |  |  |  |  |
| Yes | 111 (37%) | 15 (21%) | 45 (45%) | 24 (30%) | 27 (56%) |
| No | 103 (34%) | 42 (58%) | 15 (15%) | 33 (41%) | 13 (27%) |
| Sometimes | 85 (28%) | 15 (21%) | 38 (38%) | 24 (30%) | 8 (17%) |
|  |  |  |  |  |  |
| **Access to resources to support in caring for women during childbirth (equipment and medication)** |  |  |  |  |  |
| Yes | 195 (65%) | 35 (49%) | 71 (70%) | 59 (73%) | 30 (63%) |
| No | 33 (11%) | 23 (32%) | 3 (3%) | 5 (6%) | 2 (4%) |
| Sometimes | 71 (24%) | 14 (19%) | 24 (24%) | 17 (21%) | 16 (33%) |
|  |  |  |  |  |  |
| **Informed about changes in hospital which affect practice** |  |  |  |  |  |
| Yes | 167 (55%) | 30 (42%) | 53 (52%) | 48 (59% | 36 (75%) |
| No | 63 (21%) | 29 (40%) | 13 (13%) | 16 (20%) | 5 (10%) |
| Sometimes | 39 (23%) | 13 (18%) | 32 (32%) | 17 (21%) | 7 (15%) |
|  |  |  |  |  |  |
| **Feeling supported by their manager** |  |  |  |  |  |
| Always | 111 (37%) | 7 (10%) | 38 (38%) | 35 (43%) | 31 (65%) |
| Sometimes | 139 46%) | 36 (50%) | 47 (47%) | 39 (48%) | 17 (35%) |
| Don’t know | 14 (5%) | 12 (17%) | 2 (2%) | none | none |
| Rarely | 25 (%) | 10 (14%) | 8 (8%) | 7 (9%) | none |
| Never | 10 (3%) | 7 (10%) | 3 (3%) | none | none |
|  |  |  |  |  |  |
| **Received training on how to recognize or assess symptoms of COVID-19 among women** |  |  |  |  |  |
| Yes | 129 (43%) | 17 (24%) | 66 (65%) | 17 (21%) | 29 (60%) |
| No | 169 (56%) | 55 (76%) | 32 (32%) | 63 /(%) | 19 (40%) |
|  |  |  |  |  |  |
| **Training has been provided on caring for a woman who is suspected of or has COVID-19** |  |  |  |  |  |
| Yes | 99 (44%) | 8 (11%) | 50 (50%) | 20 (25%) | 21 (44%) |
| No | 200 (66%) | 64 (89%) | 48 (48%) | 61 (75%) | 27 (56%) |
|  |  |  |  |  |  |
| **Personal protective equipment is available to staff in sufficient quantity to change between patients** |  |  |  |  |  |
| Yes | 76 (25%) | 10 (14%) | 28 (28%) | 22 (27%) | 16 (33%) |
| No | 156 (52%) | 54 (75%) | 30 (30%) | 50 (62%) | 22 (46%) |
| Sometimes | 67 (22%) | 8 (11%) | 40 (40%) | 9 (11%) | 10 (21%) |

| **Survey section 3. Triage and referral** |  |  |  |  |  |
| --- | --- | --- | --- | --- | --- |
|  | **All countries** | **Benin** | **Malawi** | **Tanzania** | **Uganda** |
|  | (n = 302) (%) | (n= 72) (%) | (n=101) (%) | (n=81) (%) | (n=48) (%) |
| **The facility has a written protocol in place for triage related to labour and childbirth** (n=301) |  |  |  |  |  |
| Yes | 163 (54) | 18 (25) | 52 (51) | 55 (68) | 38 (79) |
| No | 113 (37) | 51 (71) | 31 (31) | 22 (27) | 9 (19) |
| Don't know | 24 (8) | 3 (4) | 16 (16) | 4 (5) | 1 (2) |
|  |  |  |  |  |  |
| **The facility has a written protocol in place for referral during labour and childbirth (only for non-referral facilities)** (n=299) |  |  |  |  |  |
| Yes | 135 (45) | 13 (18) | 45 (45) | 56 (69) | 21 (44) |
| No | 61 (20) | 22 (31) | 14 (14) | 10 (12) | 15 (31) |
| Don’t know | 23 (8) | 5 (7) | 11 (11) | 4 (5) | 3 (6) |
| Working in referral hospital | 80 (26) | 32 (44) | 28 (28) | 11 (14) | 9 (19) |
|  |  |  |  |  |  |
| **The facility has a written protocol in place for the assessment of women with symptoms of COVID-19** (n=301) |  |  |  |  |  |
| Yes | 106 (35) | 15 (21) | 50 (50) | 22 (27) | 19 (40) |
| No | 152 (50) | 48 (67) | 32 (32) | 48 (59) | 24 (50) |
| Don’t know | 42 (14) | 9 (12) | 17 (17) | 11 (14) | 5 (10) |

| **Survey section 4. First stage management of labour** | | |  |  |  |
| --- | --- | --- | --- | --- | --- |
|  | **All countries** | **Benin** | **Malawi** | **Tanzania** | **Uganda** |
|  | (n = 302) (%) | (n= 72) (%) | (n=101) (%) | (n=81) | (n=48) |
| **Partograph is used if available** |  |  |  |  |  |
| Yes | 278 (92) | 61 (85) | 96 (95) | 76 (94) | 45 (94) |
| No | 12 (4) |  | 4 (4) | 5 (6) | 3 (6) |
| No response | 11 (4) | 11 (15) |  |  |  |
|  |  |  |  |  |  |
| **Recommendation to women regarding eating and drinking** |  |  |  |  |  |
| Encourage to drink and eat as wanted | 188 (62) | 31 (18) | 62 (61) | 63 (78) | 32 (67) |
| Restrict food but encourage to drink | 76 (25) | 13 (18) | 30 (30) | 17 (21) | 16 (33) |
| Encourage food but restrict drink | 11 (4) | 5 (7) | 5 (5) | 1 (1) |  |
| Restrict both to drink and eat | 18 (6) | 17 (24) | 1 (1) |  |  |
| Abstain fully from drinking and eating | 7 (2) | 6 (8) | 1 (1) |  |  |
|  |  |  |  |  |  |
| **Management of companionship when the woman is in labour and during birth** |  |  |  |  |  |
| Encourage strongly throughout labour and birth (1st and 2nd stage) | 175 (58) | 46 (64) | 74 (73) | 13 (16) | 42 (88) |
| Encourage during labour (1st stage) but discourage during birth (2nd stage) | 39 (13) | 10 (14) | 8 (8) | 16 (20) | 5 (10) |
| Discourage during labour (1st stage) but encourage during birth (2nd stage) | 4 (1) |  | 2 (2) | 1 (1) | 1 (2) |
| Do not encourage as not important according to my view | 17 (6) | 2 (3) | 1 (1) | 14 (17) |  |
| Do not encourage as not policy at this facility | 66 (22) | 14 (19) | 15 (15) | 37 (46) |  |
|  |  |  |  |  |  |
| **How often should the fetal heart rate be checked and recorded during active phase of the 1^st^ stage of labour?** |  |  |  |  |  |
| Every 15 minutes | 44 (15) | 27 (38) | 8 (8) | 7 (9) | 2 (4) |
| Every 20 minutes | 2 (1) |  | 1 (1) |  | 1 (2) |
| Every 30 minutes | 246 (81) | 40 (56) | 89 (88) | 73 (90) | 44 (92) |
| Every 60 minutes | 7 (2) | 3 (4) | 2 (2) | 1 (1) | 1 (2) |
| I don’t monitor |  |  |  |  |  |
| I don’t know | 2 (1) | 2 (3) |  |  |  |

| **Survey section 5. Second stage management of labour** | |  |  |  |  |
| --- | --- | --- | --- | --- | --- |
|  | **All countries** | **Benin** | **Malawi** | **Tanzania** | **Uganda** |
|  | (n = 301) (%) | (n= 72) (%) | (n=100) (%) | (n=81) (%) | (n=48) (%) |
| **How often should the fetal heart rate be monitored in the 2^nd^ stage?** |  |  |  |  |  |
| Every 5 minutes | 43 (14) | 7 (10) | 15 (15) | 12 (15) | 9 (14) |
| Every 15 minutes | 134 (45) | 25 (35) | 45 (45) | 35 (43) | 29 (44) |
| Every 30 minutes | 108 (36) | 37 (51) | 37 (37) | 29 (36) | 5 (36) |
| Every 60 minutes | 4 (1) | 1 (1) | 1 (1) | 1 (1) | 1 (1) |
| I don’t monitor | 5 (2) |  | 1 (1) | 2 (2) | 2 (4) |
| I don’t know | 6 (2) | 2 (3) | 1 (1) | 2 (2) | 1 (2) |
|  |  |  |  |  |  |
| **Been taught during pre-service training how to perform an episiotomy?** |  |  |  |  |  |
| Yes | 292 (97) | 72 (100) | 94 (94) | 80 (99) | 46 (96) |
| No | 9 (3) |  | 6 (6) | 1 (1) | 2 (4) |
|  |  |  |  |  |  |
| **Using/applying anaesthesia for suturing an episiotomy?** |  |  |  |  |  |
| Yes | 289 (96) | 67 (93) | 96 (96) | 80 (99) | 46 (96) |
| No | 12 (4) | 5 (7) | 4 (4) | 1 (1) | 2 (4) |
|  |  |  |  |  |  |
| **Under most circumstances, when should a woman be supported to begin breastfeeding?** |  |  |  |  |  |
| After the baby’s first bath | 3 (1) | 3 (4) |  |  |  |
| When the baby first starts to cry | 10 (3) | 1 (1) | 6 (6) | 3 (4) |  |
| As soon as possible when the baby is ready within the first hour after birth | 286 (95) | 68 (95) | 94 (94) | 76 (94) | 48 (100) |
| When her milk comes in | 2 (1) |  |  | 2 (3) |  |
|  |  |  |  |  |  |
| **What does APGAR stands for?** |  |  |  |  |  |
| Correct | 175 (58) | 24 (33) | 58 (58) | 52 (64) | 41 (85) |
| Partly correct | 88 (29) | 21 (29) | 34 (34) | 26 (32) | 7 (15) |
| Don't know | 27 (9) | 27 (38) |  |  |  |
| Missing | 11 (4) |  | 8 (8) | 3 (3) |  |

| **Survey section 5. Second stage management of labour - questions with multiple response options** | | | | | | | | | | |
| --- | --- | --- | --- | --- | --- | --- | --- | --- | --- | --- |
|  | **All countries** (n=301) | | **Benin** (n=72) | | **Malawi** (n=100) | | **Tanzania** (n=81) | | **Uganda** (n=48) | |
|  | **Number of times option chosen** | **% among participants** | **Number of times option chosen** | **% among participants** | **Number of times option chosen** | **% among participants** | **Number of times option chosen** | **% among participants** | **Number of times option chosen** | **% among participants** |
| **What are the benefits of giving birth while standing, squatting, or kneeling?** |  |  |  |  |  |  |  |  |  |  |
| Shorter second stage | 134 | 45 | 38 | 53 | 53 | 53 | 23 | 28 | 20 | 42 |
| Less blood loss | 11 | 4 | 5 | 7 | 1 | 1 | 4 | 5 | 1 | 2 |
| Lower risk of second-degree tears | 69 | 23 | 16 | 22 | 27 | 27 | 10 | 12 | 16 | 33 |
| I did not learn about a benefit | 132 | 44 | 30 | 42 | 31 | 31 | 51 | 63 | 20 | 42 |
|  |  |  |  |  |  |  |  |  |  |  |
| **When do you decide to intervene during the second stage of labour?** |  |  |  |  |  |  |  |  |  |  |
| When the second stage extends beyond the standard duration | 234 | 78 | 62 | 86 | 84 | 84 | 51 | 63 | 37 | 77 |
| When there is fetal distress | 163 | 54 | 60 | 83 | 44 | 44 | 30 | 37 | 29 | 60 |
| When there is evidence of progress in the descent of the fetal head | 78 | 26 | 14 | 19 | 19 | 19 | 35 | 43 | 10 | 21 |
|  |  |  |  |  |  |  |  |  |  |  |
| **Immediate care for a normal newborn includes the following actions?** |  |  |  |  |  |  |  |  |  |  |
| Stimulating the baby by slapping the soles of the baby’s feet | 65 | 22 | 29 | 40 | 12 | 12 | 17 | 21 | 7 | 15 |
| Drying the baby | 225 | 75 | 51 | 71 | 79 | 78 | 51 | 63 | 44 | 92 |
| Placing the baby in a baby warmer | 91 | 30 | 28 | 39 | 20 | 20 | 27 | 33 | 16 | 33 |
| Placing the baby skin-to-skin with the mother | 266 | 88 | 70 | 97 | 80 | 79 | 75 | 93 | 41 | 85 |

| **Survey section 6. Third stage management of labour** | |  |  |  |  |
| --- | --- | --- | --- | --- | --- |
|  | **All countries** | **Benin** | **Malawi** | **Tanzania** | **Uganda** |
|  | (n = 302) | (n= 72) | (n=101) | (n=81) | (n=48) |
| **In the first two hours after giving birth how often should a women be monitored (uterine tone, bleeding, BP, and pulse)?** |  |  |  |  |  |
| Every 5 minutes | 15 (5) | 3 (4) | 3 (3) | 3 (4) | 6 (13) |
| Every 15 minutes | 191 (63) | 65 (90) | 69 (68) | 37 (46) | 20 (42) |
| Every 30 minutes | 96 (32) | 4 (6) | 29 (29) | 41 (51) | 22 (46) |
|  |  |  |  |  |  |
| **If the baby is crying and does not need resuscitation, when should you clamp or tie the umbilical cord?** |  |  |  |  |  |
| Immediately after birth | 106 (35) | 34 (47) | 24 (24) | 32 (40) | 16 (33) |
| 1 to 3 minutes after birth | 177 (59) | 34 (47) | 71 (70) | 42 (52) | 30 (63) |
| 5 minutes after birth | 19 (6) | 4 (6) | 6 (6) | 7 (9) | 2 (4) |

| **Survey section 6. Third stage management of labour - questions with multiple response options** | | | | | | |  |  |  |  |
| --- | --- | --- | --- | --- | --- | --- | --- | --- | --- | --- |
|  | **All countries** (n=301) | | **Benin** (n=72) | | **Malawi** (n=100) | | **Tanzania** (n=81) | | **Uganda** (n=48) | |
|  | **Number of times option chosen** | **% among participants** | **Number of times option chosen** | **% among participants** | **Number of times option chosen** | **% among participants** | **Number of times option chosen** | **% among participants** | **Number of times option chosen** | **% among participants** |
| **How do you monitor blood loss during the third stage?** |  |  |  |  |  |  |  |  |  |  |
| Visual estimation of blood loss (e.g. counting the number of saturated pads) | 272 | 90 | 59 | 82 | 93 | 92 | 74 | 91 | 46 | 96 |
| Pulse rate and blood pressure measurement | 141 | 47 | 42 | 58 | 49 | 49 | 30 | 37 | 20 | 42 |
| Hematocrit (red blood cell count) | 55 | 18 | 22 | 31 | 11 | 11 | 16 | 20 | 6 | 13 |
| I compare to other births where I have provided care | 8 | 3 | 2 | 3 | 1 | 1 |  |  | 5 | 10 |
| Other | 22 | 7 | 8 | 11 | 6 | 6 | 6 | 7 | 2 | 4 |
|  |  |  |  |  |  |  |  |  |  |  |
| **Common signs and symptoms of postpartum preeclampsia** |  |  |  |  |  |  |  |  |  |  |
| High blood pressure, usually over 140/90 | 283 | 94 | 72 | 100 | 86 | 85 | 78 | 96 | 47 | 98 |
| High levels of protein in the urine | 233 | 77 | 69 | 96 | 78 | 77 | 50 | 62 | 36 | 75 |
| Cold hands and feet | 5 | 2 | 1 | 1 | 4 | 4 |  |  |  |  |

| **Survey section 7. Reporting and documentation and handover between shifts** | | | |  |  |
| --- | --- | --- | --- | --- | --- |
|  | **All countries** | **Benin** | **Malawi** | **Tanzania** | **Uganda** |
|  | (n = 302) (%) | (n= 72) (%) | (n=101) (%) | (n=81) (%) | (n=48) (%) |
| **For reporting and documentation which format is used?** |  |  |  |  |  |
| Paper format (book) | 222 (74) | 65 (90) | 76 (75) | 40 (49) | 41 (85) |
| Electronic format (computer) | 4 (4) |  | 2 (2) | 2 (2) |  |
| Both paper and electronic format | 76 (76) | 7 (7) | 23 (23) | 39 (48) | 7 (15) |
|  |  |  |  |  |  |
| **Do you know how much time is approximately allocated to handover between shifts?** |  |  |  |  |  |
| Yes | 184 (61) | 42 (58) | 39 (39) | 68 (84) | 35 (73) |
| No | 118 (39) | 30 (42) | 62 (61) | 13 (16) | 13 (27) |
|  |  |  |  |  |  |
| **If yes, approximate number of minutes allocated to handover between shifts** | Figures implausible - It was reported that handover varies from 1 to 730 minutes. | | | | |

| **Survey section 7. Reporting and documentation and handover between shifts - question with multiple response options** | | | | | | | | |  |  |
| --- | --- | --- | --- | --- | --- | --- | --- | --- | --- | --- |
|  | **All countries** (n=301) | | **Benin** (n=72) | | **Malawi** (n=100) | | **Tanzania** (n=81) | | **Uganda** (n=48) | |
|  | **Number of times option chosen** | **% among participants** | **Number of times option chosen** | **% among participants** | **Number of times option chosen** | **% among participants** | **Number of times option chosen** | **% among participants** | **Number of times option chosen** | **% among participants** |
| **Why is it needed to document and report information?** |  |  |  |  |  |  |  |  |  |  |
| The health managers need the data regularly | 169 | 56 | 62 | 86 | 43 | 43 | 35 | 43 | 29 | 60 |
| My colleagues need to know about the women | 209 | 69 | 37 | 51 | 72 | 71 | 64 | 79 | 36 | 75 |
| Data are used for quality improvement | 267 | 88 | 66 | 92 | 96 | 95 | 64 | 79 | 41 | 85 |
| The women need the information | 86 | 28 | 26 | 36 | 29 | 29 | 18 | 22 | 13 | 27 |
