# Supplementary material for: Midwifery care providers’ childbirth and immediate newborn care competencies: A cross-sectional study in Benin, Malawi, Tanzania and Uganda
Source: PLOS Glob Public Health. 2023 Jun 6;3(6):e0001399. doi: 10.1371/journal.pgph.0001399 (PMC10243614; doi:10.1371/journal.pgph.0001399)
Supplement: S1 Text — (DOCX) [file pgph.0001399.s005.docx]

**S1 Text. Knowledge assessment questionnaire**

**Unique identifier for health facilities**

• Uganda: UG (hospital; UG/01, UG/02, UG/03, UG/04)

• Malawi: MW (hospital; MW/01, MW/02, MW/03, MW/04)

• Benin: BJ (hospital; BJ/01, BJ/02, BJ/03, BJ/04)

• Tanzania: TZ (hospital; TZ/01, TZ/02, TZ/03, TZ/04)

**Unique identifier for health care providers:** H for any health care provider

**Tool WP3: 3/1 (part 1 of 2)**

Country 

Hospital 

Tool /

Type H

Participant ####

////H/##

**Supervisor: Name** (capital letters) _________________ **Signature___________________ Date: ___/___/**

**We would like to ask you some questions about your education, workplace, skills, knowledge and competencies related to the care you provide to women in labour and childbirth in your hospital. The information will be used to inform the content of the in-service training programme which will be conducted in your hospital as part of the ALERT project.**

**Please follow the instruction indicated under each question.**

**Date (dd/mm/year/): ___/___/______**

**Section 1. Provider characteristics (personnel information, educational background and general information related to current job)**

| **No** | **Variable name** | **Variable/Questions** | **Values/Answer option** | **Instructions** | **Comments** |
| --- | --- | --- | --- | --- | --- |
| 1 | s1q1 | What is your sex? (please tick one answer) | 1. Male  2. Female |  |  |
| 2 | s1q2_B | What is your highest school education? (please tick one answer) | 1. Completed primary school  2. Completed ordinary secondary school  3. Completed advanced secondary school |  |  |
|  | s1q2_M | What is your highest school education? (please tick one answer | 1. Completed primary school  2. Completed ordinary secondary school  3. Completed advanced secondary school |  |  |
|  | s1q2_T | What is your highest school education? (please tick one answer | 1. Completed primary school  2. Completed ordinary secondary school  3. Completed advanced secondary school |  |  |
|  | s1q2_U | What is your highest school education? (please tick one answer | 1. Completed primary school  2. Completed ordinary secondary school  3. Completed advanced secondary school |  |  |
| 3 | s1q3_B | What is your highest degree?  (please tick) | 1. Certificate  2. Bachelor  3. Master  4. Doctoral degree |  |  |
|  | s1q3_M | What is your highest degree?  (please tick) | 1. Certificate  2. Diploma  3. Bachelor  4. Master  5. Doctoral degree |  |  |
|  | s1q3_T | What is your highest degree?  (please tick) | 1. Certificate  2. Diploma  3. Bachelor  4. Master  5. Doctoral degree |  |  |
|  | s1q3_U | What is your highest degree?  (please tick) | 1. Certificate  2. Diploma  3. Bachelor  4. Master  5. Doctoral degree |  |  |
| 4 | s1q4 | How many months/years were you in total in pre-service training in nursing/midwifery/clinical science? (please write) | 1. Less than 2 years  2. More than 2 years |  | **Please indicate months if less than 2 years, otherwise entre years.** |
| 5 | s1q5 | How many months of pre-service training did you receive specifically in midwifery care? (please write) | 1. Please write number of months: |  |  |
| 6 | s1q6_B | What is your professional title? (please tick one answer) | 1. Nursing auxiliary  2. Midwife  3. Nurse  4. Doctor  5. Other |  | **If other, please write.** |
|  | s1q6_M | What is your professional title? (please tick one answer) | 1. Maid  2. Midwife  3. Nurse  4. Doctor  5. Other |  | **If other, please write.** |
|  | s1q6_T | What is your professional title? (please tick one answer) | 1. Midwife  2. Nurse  3. Doctor  4. Other |  | **If other, please write.** |
|  | s1q6_U | What is your professional title? (please tick one answer) | 1. Midwife  2. Nurse  3. Doctor  4. Other |  | **If other, please write.** |
| 7 | s1q7_B | What is your title in your current job? (cadre name) (please tick one answer) | 1. Registered Midwife  2. Registered Nurse  3.Doctor  4. Other |  | **If other, please write.** |
|  | s1q7_M | What is your title in your current job? (cadre name) (please tick one answer) | 1. Maid  2. Nurse-midwife technician  3. Registered nurse-midwife  4. Nurse Officer  5. Intern Clinical Technician  6. Clinical Technician  7. Clinical Officer  8. Medical Officer  9. Senior Medical Officer  10. Health Surveillance Assistant  11. Other |  | **If other, please write.** |
|  | s1q7_T | What is your title in your current job? (cadre name) (please tick one answer) | 1. Enrolled nurse-midwife  2. Registered nurse-midwife  3. Medical Assistant  4. Assistant Medical Officer  5. Medical Officer  6. Other |  | **If other, please write.** |
|  | s1q7_U | What is your title in your current job? (cadre name) (please tick one answer) | 1. Enrolled midwife  2. Registered midwife  3. Intern Nurse  4. Intern Doctor  5. Clinical Officer  6. Medical Officer  7. Obstetrician  8. Gynaecologist  9. Other |  | **If other, please write.** |
| 8 | s1q8 | How many year(s) of experience do you have in providing care to women giving birth? (please indicate number year(s)) (please write) | Please write the number of whole year(s) experience you have:  (Please exclude the periods you have been on maternity/paternity leave or long-term sick leave). |  |  |
| 9 | s1q9 | What kind of shift do you typically do? (please tick) | 1. Day  2. Evening  3. Night  4. Rotating shifts |  |  |
| 10 | s1q10 | Approximately, how many births did you conduct in the last month?  (please write) | 1. Please write the number of births: |  |  |

**Section 2. Working environment (in-service training)**

| **No** | **Variable Name** | **Variable/Questions** | **Values/Answer option** | **Instructions** | **Comments** |
| --- | --- | --- | --- | --- | --- |
| 1 | s2q1 | Are you registered with a professional association/organization? (please tick) | 1. Yes  2. No  3. I don’t know |  | If yes, please write which organization: |
| 2 | s2q2 | Do you receive any supervision for your work related to labour and childbirth? (please tick) | 1. Yes  2. No  3. Sometimes |  | If yes, please describe how the supervision is provided: |
| 3 | s2q3 | Do you have access to resources for staff education and training? | 1. Yes 2. No 3. Sometimes |  |  |
| 4 | s2q4 | Do you have access to resources to support you in caring for labouring women? | 1. Yes 2. No 3. Sometimes |  |  |
| 5 | s2q5 | Are you informed about changes in your hospital that will affect your practice? | 1. Yes 2. No 3. Sometimes |  |  |
| 6 | s2q6 | Do you feel supported by your manager? | 1. Always 2. Sometimes 3. Don’t know 4. Rarely 5. Never |  |  |
| 7 | s2q7 | Have you had any training on how to recognize or assess symptoms of COVID-19 among women? | 1. Yes 2. No |  |  |
| 8 | s2q8 | Have you had any training on caring for a woman who is suspected of or has COVID-19? | 1. Yes 2. No |  |  |
| 9 | s2q9 | Is personal protective equipment available to you in sufficient quantity to change between patients? | 1. Yes 2. No 3. Sometimes |  |  |

**Section 3. Triage and referral**

| **No** | **Variable name** | **Variable/Questions** | **Values/Answer option** | **Instructions** | **Comments** |
| --- | --- | --- | --- | --- | --- |
| 1 | s3q1 | In your facility is there a written protocol in place for triage related to labour and birth? (please tick) | 1. Yes  2. No  3. I don’t know |  |  |
| 2 | s3q2 | Does your facility have a written protocol in place for referral during labour and birth? Only for non-referral hospitals (please tick) | 1. Yes  2. No  3. I don’t know  4. I work in a referral hospital |  |  |
| 3 | s3q3 | Does your facility have a written protocol in place for the assessment of women with symptoms of COVID-19? | 1. Yes 2. No 3. I don’t know |  |  |

**Section 4. First stage management of labour**

| **No** | **Variable name** | **Variable/Questions** | **Values/Answer option** | **Instructions** | **Comments** |
| --- | --- | --- | --- | --- | --- |
| 1 | s4q1 | If it is available, do you always use a partograph? (please tick) | 1. Yes  2. No |  |  |
| 2 | s4q2 | Concerning eating and drinking, what do you typically recommend? (please tick) | 1. Encourage to drink and eat as wanted.  2. Restrict food but encourage to drink.  3. Encourage food but restrict drink  4. Restrict both to drink and eat.  5. Abstain fully from drinking and eating. |  |  |
| 3 | s4q3 | How do you typically manage companionship, which is that the women in labour and childbirth has someone she knows with her and supporting her? (please tick) | 1. Encourage strongly throughout labour and childbirth (1st and 2nd stage).  2. Encourage during labour (1^st^ stage) but discourage during birth (2nd stage).  3. Discourage during labour (1^st^ stage) but encourage during birth (2^nd^ stage)  4. Do not encourage as not important according to my view.  5. Do not encourage as not policy at this facility. |  |  |
| 4 | s4q4 | According to what you were taught, how often should the fetal heart rate be checked and recorded during active phase of the 1^st^ stage of labour? (please tick one answer) | 1. Every 15 minutes  2. Every 20 minutes  3. Every 30 minutes  4. Every 60 minutes  5. I don’t monitor  6. I don’t know |  |  |

**Section 5. Second stage management of labour**

| **No** | **Variable name** | **Variable/Questions** | **Values/Answer option** | **Instructions** | **Comments** |
| --- | --- | --- | --- | --- | --- |
| 1 | s5q1 | According to what you were taught, how often should the fetal heart rate be monitored in the 2^nd^ stage? (please tick one answer) | 1. Every 5 minutes  2. Every 15 minutes  3. Every 30 minutes  4. Every 60 minutes  5. I don’t monitor  6. I don’t know |  |  |
| 2 | s5q2 | According to what you were taught, what are the benefits of giving birth while standing, squatting, or kneeling? (please tick all answers that apply) | 1. Shorter second stage.  2. Less blood loss.  3. Lower risk of second-degree tears.  4. I did not learn about a benefit. |  |  |
| 3 | s5q3 | According to what you were taught, when do you decide to intervene during the second stage of labour? (please tick all that apply) | 1. When the second stage extends beyond the standard duration.  2. When there is fetal distress.  3. When there is evidence of progress in the descent of the fetal head. |  |  |
| 4 | s5q4 | Were you taught in your pre-service training how to perform an episiotomy? (please tick) | 1. Yes  2. No |  |  |
| 5 | s5q5 | Do you use/apply anaesthesia for suturing an episiotomy? | 1. Yes  2. No |  |  |
| 6 | s5q6 | Immediate care for a normal newborn includes which of the following actions? (please tick all that apply) | 1. Stimulating the baby by slapping the soles of the baby’s feet.  2. Drying the baby.  3. Placing the baby in a baby warmer  4. Placing the baby skin-to-skin with the mother. |  |  |
| 7 | s5q7 | Under most circumstances, when should a woman be supported to begin breastfeeding? (please tick one item) | 1. After the baby’s first bath.  2. When the baby first starts to cry.  3. As soon as possible when the baby is ready within the first hour after birth.  4. When her milk comes in. |  |  |

**Section 6. Third stage management of labour**

| **No** | **Variable Name** | **Variable/Questions** | **Values/Answer option** | **Instructions** | **Comments** |
| --- | --- | --- | --- | --- | --- |
| 1 | s6q1 | How often should you monitor a woman (uterine tone, bleeding, BP, and pulse) in the first two hours after giving birth? (please tick one answer) | 1. Every 5 minutes  2. Every 15 minutes  3. Every 30 minutes |  |  |
| 2 | s6q2 | In your daily work, how do you monitor blood loss during the third stage? (please tick all that apply) | 1. Visual estimation of blood loss (e.g., counting the number of saturated pads).  2. Pulse rate and blood pressure measurement.  3. Hematocrit (red blood cell count).  4. I compare to other births where I have provided care.  5. Other |  |  |
| 3 | s6q3 | What are the common signs and symptoms of postpartum preeclampsia (please tick all that apply)? | 1. High blood pressure, usually over 140/90.  2. High levels of protein in the urine.  3. Cold hands and feet. |  |  |
| 4 | s6q4 | If the baby is crying and does not need resuscitation, when should you clamp or tie the umbilical cord? (please tick one answer) | 1. Immediately after birth.  2. 1 to 3 minutes after birth.  3. 5 minutes after birth. |  |  |
| 5 | s6q5 | What does APGAR stand for?  (please write what each letter stands for) | 1. A:  2. P:  3. G:  4. A:  5. R: |  |  |

**Section 7. Reporting and documentation, and handover between shifts**

| **No** | **Variable Name** | **Variable/Questions** | **Values/Answer option** | **Instructions** | **Comments** |
| --- | --- | --- | --- | --- | --- |
| 1 | s7q1 | Why do you think you need to document and report information about the women and their newborns as well as the care you provided? (please tick all that apply) | 1. The health managers need the data regularly.  2. My colleagues need to know about the women.  3. Data are used for quality improvement.  4. The women need the information. |  |  |
| 2 | s7q2 | For reporting and documentation which format are you using? (please tick) | 1. Paper format (book).  2. Electronic format (computer).  3. Both paper and electronic format. |  |  |
| 3 | s7q3 | How much time is allocated to handover between shifts? (please indicate approximate minutes) | 1. Minutes: **_ _**  2. I don’t know |  |  |
