# Supplementary material for: Midwifery care providers’ childbirth and immediate newborn care competencies: A cross-sectional study in Benin, Malawi, Tanzania and Uganda
Source: PLOS Glob Public Health. 2023 Jun 6;3(6):e0001399. doi: 10.1371/journal.pgph.0001399 (PMC10243614; doi:10.1371/journal.pgph.0001399)
Supplement: S2 Text — (DOCX) [file pgph.0001399.s006.docx]

**S2 Text. Skills drills script and observation checklist**

**Skills drills script**

**Introduction**

In the event that one of the midwifery care providers asks you as Mary about any of the following, please provide the answer as detailed in this checklist.

During the skills drills session please also ask the following questions:

1. During the admission assessment ask the provider “Can I have something to eat and drink?”
2. During the birth (prior to the baby being born), please ask the provider “Is my baby ok?”

**Section 2: Admission**

| Vaginal bleeding | None |
| --- | --- |
| Loss of fluid from vagina | Yes – started losing fluid approximately one hour ago  (If the provider asks it is clear and has no offensive smell) |
| Convulsions | None |
| Severe headache or blurred vision | None |
| Severe abdominal pain | None – only labour like pains |
| Respiratory difficulty | None |
| Fever | None |

**Section 3: Clinical history**

| Age | 28 |
| --- | --- |
| **Obstetric history** | |
| Number of pregnancies | This is my 3^rd^ pregnancy |
| Number of abortions | None |
| Number of normal births | Two |
| Number of caesarean sections | None |
| Number of children born alive and still alive | Two |
| Number of stillbirths | None |
| Any maternal complication in previous pregnancy | None |
| Date and outcome of last pregnancy | 24.05.2018 SVD, livebirth no maternal or neonatal complications |
| **General medical problems** | |
| Any medication | None |
| Use of alternative medication or herbs | No |
| Receiving treatment for TB/HIV | No – HIV Negative |
| **Gestational Age** | |
| Last menstrual period (LMP) | The data collector will tell you prior to the skills drills session what the date of Mary’s LMP should be |
| Expected date of delivery | The data collector will tell you prior to the skills drills session what Mary’s expected date of delivery should be |
| When did painful regular contractions start? | Contractions started 5 hours ago, but they have only been regular for 2 hours |
| Frequency and strength of contractions | 3 contractions in 10 minutes, lasting 50 seconds |
| **Waters/membranes rupturing** | |
| When waters broke | One hour ago |
| What colour was the water | Clear |
| What smell did the waters have | None |
| Are you feeling baby’s movements | Yes – movements are in a normal pattern |

**Section 4: Physical examination**

| Temperature | 36.8C |
| --- | --- |
| Blood pressure | 122/76 |
| Pulse | 81 |
| Respiratory rate | 18 |
| Oedema | Mild oedema in the feet and ankles |
| Conjunctiva | Normal – no signs of anaemia |

**Section 5: Obstetric examination**

| Abdominal appearance | NAD – no scars noted |
| --- | --- |
| Fundal height  Fetal lie  Presentation  Engagement | 38cm  Longitudinal  Cephalic  2/5^th^ palpable |
| Contractions | 3:10 minutes, 50 seconds long, moderate to strong on palpation, good resting tone between |
| Auscultates FH | 138 bpm |

**Section 6: Vaginal examination**

| Length  Texture  Dilation | <1cm long (fully effaced)  Soft and stretchy  8cm |
| --- | --- |
| Assess for membranes  Check for cord prolapse | Ruptured and clear liquor draining  No evidence of cord prolapse |
| Level of the presenting part | At spines -1 |
| Fetal presentation  Fetal position | Cephalic  LOA |

**Section 8: The provider assists the woman to have a clean, safe birth**

| FH Rate during second stage of labour | If the provider monitors the FH during the second stage of labour, please choose a number between 130-140 each time they listen, and tell them that this is the heart rate. |
| --- | --- |

**How to brief the person playing Mary**

The person playing Mary must be appropriately prepared to play the role. Please provide the person playing Mary with the script, and explain that they will be expected to use this to answer any of the questions that the provider may ask them.

On the day of the skills drills session, you as the data collector, need to give the person playing Mary a date for Mary’s LMP and EDD. The scenario states that Mary is 39 weeks pregnant, so please calculate the LMP and EDD to reflect this.

Please also explain to the person playing Mary that:

- If the provider conducts any assessments/examinations (e.g. pulse, blood pressure, abdominal palpation, vaginal examination), that they should tell the provider the result of the assessment using the information in the script.
- It is important for them to act as if they are in labour. In particular, this means that they act as if they are having three contractions in every 10 minute period. They therefore must act as if they are in pain.

**Skills drills session equipment list**

- Bed
- Mamabirthie kit
- Blood pressure cuff and stethoscope
- Thermometer
- Clock/watch
- Handwashing facilities including soap/towel
- Tape measure
- Pinnard stethoscope/fetal heart rate doppler
- Gloves
- Apron
- Boots (if necessary)
- Delivery pack including cord clamps, 2 pairs of scissors (one to cut the cord, another to perform an episiotomy if necessary).
- Cloths or towels to dry and wrap the baby at birth
- Cloths to cover the mother
- Vial of oxytocin (10 iu)
- Needles and syringes
- Sharps disposal box
- Waste bin
- Bulb syringe
- Baby weighing scale
- Tetracycline eye ointment
- Vitamin K
- Light for checking the perineum
- Pad for the mother after birth
- Paperwork for documentation

Equipment required for data collectors

- Paper copies of the ALERT participant information leaflet
- Paper copies of consent form
- Paper copies of observation tool
- Paper copy of SOP
- List of unique identifying numbers
- Tablet
- Pen/pencil
- REDCap Manual

**Guidance on completing the observation checklist**

As the data collector you will introduce the skills drills session. To introduce the skills drills session please welcome the particpant. Explain that you will present two scenarios to the participant and then ask them to act out the care that they would provide in each scenario.

Scenario One: To introduce the first scenario please explain that Mary is a 28-year-old P2 who is 39 weeks pregnant and has just presented at the health care facility with a history of contractions for the past five hours, which have been regular for the past two hours. Mary also reports that her waters have broken. Then ask the participant being observed to perform an initial assessment of Mary. Explain to the participant that they may verbalise what they are doing so that the data collector is able to assess skills that the participant may not be able to physically demonstrate, for example, the participant may say “I am looking at the perineum as the head is advancing so that I can assess if an episiotomy is necessary”.

**As you observe the practice of the midwifery care provider you should complete the checklist as follows:**

If the intrapartum provider carries out the defined task mark the checklist with “Done”

If the intrapartum provider does not carry out the defined task, mark the checklist with “Not Done”

If it is not applicable for the intrapartum provider to carry out the defined task, mark the checklist “Not applicable”

If you make any other observations or have any additional comments to make about the care provided by the intrapartum care provider, there will be a free text box available at the end of the checklist for you to make these comments.

Once the participant has completed the initial assessment of Mary, you as the data collector, should introduce the second scenario:

Mary’s labour has progressed normally and she is now feeling rectal pressure and an urge to push. Assume that Mary is fully dilated. Please demonstrate the care that you would provide for Mary from this point until 15 minutes following the birth of the baby.

**Again, as you observe the practice of the midwifery care provider you should complete the checklist as follows:**

If the intrapartum provider carries out the defined task mark the checklist with “Done”

If the intrapartum provider does not carry out the defined task, mark the checklist with “Not Done”

If it is not applicable for the intrapartum provider to carry out the defined task, mark the checklist with “Not applicable”

If you make any other observations or have any additional comments to make about the care provided by the intrapartum care provider, there will be a free text box available at the end of the checklist for you to make these comments.

Clarification regarding what the data collector is expected to observe:

s1q9 “Provides emotional support and reassurance” – here we are looking for the participant to offer Mary comfort during her contractions and reassurance when Mary asks any questions where she shows concern (e.g. offers kind words to support Mary during her contractions, reassures Mary that her baby is coping well with the labour).

s4q13 “Observes Mary’s emotional response to labour” – here we are looking for the participant to offer Mary comfort during her contractions and reassurance when Mary asks any questions where she shows concern (e.g. offers kind words to support Mary during her contractions, reassures Mary that her baby is coping well with the labour).

| **PERFORMANCE STANDARDS** | **Obs No.** | **Variable name** | **Verification criteria** | **Answer option (unless otherwise stated**  **1 = Done**  **0= Not done**  **9= Not applicable)** | **Additional notes** |
| --- | --- | --- | --- | --- | --- |
| **Skills drills 1_admission** | | |  | | |
| **ALERT ID** |  | alert_id | ALERT ID | The data collector should insert the ALERT ID from the prepared list of unique ID numbers |  |
| **Initial method of data collection** |  | data_coll | Where are you documenting your observations of the skills drills session? | 1. On paper  2. On a tablet |  |
| **Hospital name** |  | hosp_nam | Please select the hospital that the provider works in (Benin) | 1. Hospital de Zone Come-Grand Popo Houeyogbe Bopa  2. Centre Hospitalier et Dépt Zou-Collines  3. Centre National Hospitalier Hubert Maga  4. Policlinique Bon Samaritan |  |
|  |  | hosp_nam | Please select the hospital that the provider works in (Malawi) | 1. Mchinji Hospital  2. Ntcheu District Hospital  3. Mitundu Rural Hospital  4. St. Gabriel’s Hospital |  |
|  |  | hosp_nam | Please select the hospital that the provider works in (Tanzania) | 1. Ndanda Hospital  2. Mkomaindo Hospital  3. Nachingwea Hospital  4. Newala District Hospital |  |
|  |  | hosp_nam | Please select the hospital that the provider works in (Uganda) | 1. Jinja Regional Hospital  2. Kamuli Mission Hospital  3. Iganga Referral Hospital  4. Bugiri General Hospital |  |
| **Professional job title, qualifications and years of experience** |  | job_title | Provider’s current job title (Benin) | 1. Auxiliary Nurse  2. Registered Midwife  3. Registered Nurse  4.Doctor  5. Other |  |
|  |  | job_title | Provider’s current job title (Malawi) | 1. Health Surveillance Assistant  2. Nurse-midwife Technician  3. Registered Nurse-Midwife  4. Nurse Officer  5. Intern Clinical Technician  6. Clinical Technician  7. Clinical Officer  8. Medical Officer  9. Senior Medical Officer  10. Other |  |
|  |  | job_title | Provider’s current job title (Tanzania) | 1. Enrolled nurse-midwife  2. Registered nurse-midwife  3. Medical Assistant  4. Assistant Medical Officer  5. Medical Officer  6. Other |  |
|  |  | job_title | Provider’s current job title (Uganda) | 1. Enrolled midwife  2. Registered midwife  3. Intern Nurse  4. Intern Doctor  5. Clinical Officer  6. Medical Officer  7. Obstetrician  8. Gynaecologist  9. Other |  |
|  |  | job_titlea | If you answered other to the previous question please specify the provider’s current job title |  |  |
|  |  | high_qual | Provider’s highest degree | 1. Certificate  2. Diploma  3. Bachelor  4. Master  5. Doctoral degree |  |
|  |  | exp_years | How many completed years of experience does the provider have in providing care to women giving birth? |  |  |
| ***Skill drill scenario 1:***  ***Mary is a 28-year-old para 2***  ***She is 39 weeks pregnant and has just presented at your health care facility with a history of contractions for the past 5 hours, which have been regular for the past 2 hours. Mary also reports that her waters have broken. Please perform an initial assessment of Mary.*** | | | | | |
| **Section 1.**  **The provider treats Mary in a cordial manner.** | 1 | s1q1 | Greets Mary in a cordial manner |  |  |
|  | 2 | s1q2 | Introduces her/himself to Mary |  |  |
|  | 3 | s1q3 | Ensures that she/he speaks in easy-to-understand lay language (does not use technical words) with Mary |  |  |
|  | 4 | s1q4 | Explains to Mary the assessment procedures |  |  |
|  | 5 | s1q5 | Asks for Mary’s consent before performing procedures |  |  |
|  | 6 | s1q6 | Encourages Mary to ask questions |  |  |
|  | 7 | s1q7 | Listens to what the Mary has to say |  |  |
|  | 8 | s1q8 | Responds to questions and concerns |  |  |
|  | 9 | s1q9 | Provides emotional support and reassurance |  |  |
|  | 10 | s1q10 | Responds to Mary’s immediate needs (thirst, hunger, cold/hot, need to urinate, etc.) |  |  |
| **Section 2. Admission** | 1 | s2q1 | When assessing Mary, the provider;  determines if active labour has started (fetal wellbeing, perspiration, anxiety) |  |  |
|  | Asks Mary whether she has or had: | | | | |
|  | 2 | s2q2 | Vaginal bleeding |  |  |
|  | 3 | s2q3 | Loss of fluid from vagina |  |  |
|  | 4 | s2q4 | Convulsions |  |  |
|  | 5 | s2q5 | Severe headache and blurred vision |  |  |
|  | 6 | s2q6 | Severe abdominal pain |  |  |
|  | 7 | s2q7 | Respiratory difficulty |  |  |
|  | 8 | s2q8 | Fever |  |  |
|  | 9 | s2q9 | Records the information on Mary’s clinical history |  |  |
|  | 10 | s2q10 | Communicates findings to Mary |  |  |
| **Section 3. The provider properly reviews and fills out the clinical history about Mary.** | 1 | s3q1 | Asks and records Mary’s clinical history following information in a non-judgemental way |  |  |
|  | 2 | s3q2 | Age of Mary [in this skill drill it is 28 years] |  |  |
|  | Previous obstetric history: | | | | |
|  | 3 | s3q3 | Number of pregnancies |  |  |
|  | 4 | s3q4 | Number of abortions |  |  |
|  | 5 | s3q5 | Number of normal births |  |  |
|  | 6 | s3q6 | Number of caesarean sections |  |  |
|  | 7 | s3q7 | Number of children born alive and still alive |  |  |
|  | 8 | s3q8 | Number of still births |  |  |
|  | 9 | s3q9 | Any maternal complication in any previous pregnancy |  |  |
|  | 10 | s3q10 | Date and outcome of last pregnancy |  |  |
|  | Other general medical problems (for example diabetes, hypertension, asthma etc.): | | | | |
|  | 11 | s3q11 | Any medication |  |  |
|  | 12 | s3q12 | Use of alternative medications or herbs |  |  |
|  | 13 | s3q13 | Receiving treatment for tuberculosis and/or HIV |  |  |
|  | Gestational age – ask or calculate: | | | | |
|  | 14 | s3q14 | Last menstrual period (LMP) |  |  |
|  | 15 | s3q15 | Expected date of delivery (EDD) |  |  |
|  | Contractions | | |  |  |
|  | 16 | s3q16 | Avoids asking questions during contractions |  |  |
|  | 17 | s3q17 | Asks Mary about when her labour/when the painful regular contractions began |  |  |
|  | 18 | s3q18 | Frequency and strength of the contractions |  |  |
|  | Asks about Mary’s “bag of waters”/membrane | | | | |
|  | 19 | s3q19 | When the water broke? |  |  |
|  | 20 | s3q20 | What colour the water had? |  |  |
|  | 21 | s3q21 | What smell the water had? |  |  |
|  | 22 | s3q22 | Whether Mary feels the baby’s movements |  |  |
|  | 23 | s3q23 | Records the information on clinical history form |  |  |
|  | 24 | s3q24 | Communicates findings to Mary |  |  |
| **Section 4. The provider properly conducts the physical examination between contractions.** | 1 | s4q1 | Helps Mary onto the examination table/bed |  |  |
|  | 2 | s4q2 | Ensures that Mary remains covered with her robe or clothing |  |  |
|  | 3 | s4q3 | Asks Mary to urinate |  |  |
|  | 4 | s4q4 | Tests urine for albumin and sugar |  |  |
|  | 5 | s4q5 | Washes hands thoroughly with soap and water and dries them/uses hand gel to clean hands |  |  |
|  | 6 | s4q6 | Explains each step of the examination to Mary using easy-to-understand language |  |  |
|  | Takes vital signs: | | | | |
|  | 7 | s4q7 | Temperature |  |  |
|  | 8 | s4q8 | Blood pressure (BP) |  |  |
|  | 9 | s4q9 | Pulse |  |  |
|  | 10 | s4q10 | Respiratory rate |  |  |
|  | 11 | s4q11 | Oedema |  |  |
|  | 12 | s4q12 | Checks the conjunctiva for anaemia |  |  |
|  | 13 | s4q13 | Observes Mary’s emotional response to labour |  |  |
| **Section 5. The provider properly conducts the obstetric examination between contractions.** | 1 | s5q1 | Observes the shape and size of the abdomen and checks for the presence of scars |  |  |
|  | 2 | s5q2 | Determines fundal height, fetal lie and presentation, engagement of presenting part |  |  |
|  | 3 | s5q3 | Evaluates uterine contractions (frequency, strength and duration over a 10-minute period) |  |  |
|  | 4 | s5q4 | Auscultates fetal heart rate (FHR) |  |  |
|  | 5 | s5q5 | Records the results of the obstetric examination on the clinical history form |  |  |
|  | 6 | s5q6 | Communicates findings to Mary |  |  |
| **Section 6. The provider properly conducts a vaginal examination.** | 1 | s6q1 | Puts on a clean plastic or rubber apron |  |  |
|  | 2 | s6q2 | Washes hands thoroughly with soap and water and dries them/uses hand gel to clean hands |  |  |
|  | 3 | s6q3 | Puts examination/sterile/HLD gloves on both hands |  |  |
|  | 4 | s6q4 | Asks for permission to perform a vaginal examination. |  |  |
|  | 5 | s6q5 | Examines the vulva (ulcers, blood liquid, secretion, presenting part) |  |  |
|  | 6 | s6q6 | Cleanses the perineum with non-alcoholic antiseptic solution using the hand that will not be used to perform the examination |  |  |
|  | Performs per vaginal examination following standard technique: | | | | |
|  | 7 | s6q7 | Gently inserts two lubricated fingers of the examining hand into the vagina |  |  |
|  | 8 | s6q8 | Notes the length, texture and dilatation of the cervix |  |  |
|  | 9 | s6q9 | Notes if the membranes are intact, or if they have ruptured, ensures the cord has not prolapsed |  |  |
|  | 10 | s6q10 | Measures the level of the presenting part in centimeters above or below Mary’s ischial spines |  |  |
|  | 11 | s6q11 | Identifies fetal presentation and determines fetal position by palpating the features of the presenting part (e.g. fontanelles in cephalic presentation) |  |  |
|  | 12 | s6q12 | If gloves are disposable, places them in a waste-container with a plastic liner; OR  if they are reusable, immerses them in a 0.5% chlorine solution for at least 10 minutes before transferring them for sterilization |  |  |
|  | 13 | s6q13 | Washes hands thoroughly with soap and water and dries them/uses hand gel to clean hands |  |  |
|  | 14 | s6q14 | Record the results of the vaginal examination on the clinical history form |  |  |
|  | 15 | s6q15 | Communicates findings to Mary |  |  |
| **Section 7. Documentation** | 1 | s7q1 | Record Mary’s information and assessment of findings |  |  |
|  | | | | | |
|  | | | | | |
|  | | | | | |
| **Skill drill 2_childbirth and 15 minutes postpartum** | | | | | |
| ***Scenario 2:***  ***Mary’s labour has progressed normally, and she is now feeling rectal pressure and an urge to push.***  ***Assume that Mary is fully dilated.***  ***Please demonstrate the care that you would provide for Mary from this point until 15 minutes following the birth of the baby.***  **Section 8. The provider assists the woman to have a safe and clean birth.** | 1 | s8q1 | Preparation of equipment (e.g. delivery kit, oxytocin, gloves etc.). |  |  |
|  | 2 | s8q2 | Washes hands thoroughly with soap and water and dries them/uses hand gel to clean hands |  |  |
|  | 3 | s8q3 | Puts sterile or HLD gloves on both hands |  |  |
|  | 4 | s8q4 | Puts on personal protective equipment (plastic/rubber apron) |  |  |
|  | 5 | s8q5 | Asks for permission to cleanse the perineum |  |  |
|  | 6 | s8q6 | Cleanses the perineum with water or a non-alcoholic antiseptic solution |  |  |
|  | 7 | s8q7 | Monitors FHR every five minutes during second stage |  |  |
|  | 8 | s8q8 | Supports Mary to bear down when she feels the desire in the position she chooses (does not force her to bear down) |  |  |
|  | 9 | s8q9 | Allows the head to spontaneously crown while guarding the perineum |  |  |
|  | 10 | s8q10 | After the emergence of the head, asks Mary to briefly refrain from bearing down (open mouth breathing) and explains how to perform open mouth breathing |  |  |
|  | 11 | s8q11 | Assists Mary in birthing the baby - allows spontaneous restitution and external rotation of the head without manipulation |  |  |
|  | 12 | s8q12 | Guides the baby’s head and chest in an upward curve until the posterior shoulder has emerged over the perineum |  |  |
|  | 13 | s8q13 | Holds the baby around the chest to aid the birth of the trunk and lift it towards Mary’s abdomen |  |  |
|  | 14 | s8q14 | Places the baby on a dry towel/cloth on Mary’s abdomen |  |  |
|  | 15 | s8q15 | Wipes the baby’s eyes with a clean piece of cloth |  |  |
|  | 16 | s8q16 | Waits for 1-3 minutes before clamping and cutting the cord |  |  |
|  | 17 | s8q17 | Clamps and cuts the cord using clean/sterile blade/ instrument |  |  |
|  | 18 | s8q18 | Informs Mary of the sex of her child (with same enthusiasm if male or female) |  |  |
|  | 19 | s8q19 | Assists Mary to move her child up to her chest for skin-to-skin contact |  |  |
| **Section 9. The provider properly conducts a rapid initial assessment** | 1 | s9q1 | Receives the baby using a new set of gloves |  |  |
|  | 2 | s9q2 | Dries and stimulates the baby with a clean dry towel from head to feet |  |  |
|  | 3 | s9q3 | Discards the used towel and covers the baby including the head with a clean dry towel |  |  |
|  | 4 | s9q4 | Determines the APGAR score at 1 and 5 minutes |  |  |
|  | 5 | s9q5 | Encourages “Baby Crawl” practice and immediate breastfeeding |  |  |
|  | 6 | s9q6 | Notes the date and time of birth |  |  |
| **Section 10. The provider adequately performs active management of the third stage of labour.** | 1 | s10q1 | Touches Mary’s abdomen to rule out the presence of a second baby (without stimulating contractions) |  |  |
|  | 2 | s10q2 | Give inj. Oxytocin 10 units direct I/M and wait for next contraction within one minute after birth of the baby. |  |  |
|  | 3 | s10q3 | Holds the cord along with clamp and provides controlled cord traction/sustained downward traction |  |  |
|  | 4 | s10q4 | Repeats controlled cord traction while simultaneously applying counter pressure above pubis to guard uterus (places the other hand above the level of the symphysis pubis with hand facing towards the umbilicus to provide counter traction on the uterus) |  |  |
|  | 5 | s10q5 | Applies steady tension by pulling the cord firmly and maintaining pressure (jerky movements and force must be avoided) |  |  |
|  | 6 | s10q6 | When the placenta is visible at the vulva holds the placenta with both hands, assists in the expulsion of the placenta, by turning it over in the hands, without applying traction |  |  |
|  | 7 | s10q7 | Uses a gentle and upward and downward movement or twisting action to deliver the membranes, “teasing out” the membranes |  |  |
|  | 8 | s10q8 | Checks whether the uterus is well contracted |  |  |
|  | 9 | s10q9 | Massages the uterus with one hand on a cloth over the abdomen, until the uterus contracts firmly |  |  |
|  | 10 | s10q10 | Checks the placenta on a clean table or the delivery bed with a good light source: whether all the lobules are present and fit together to see if complete (missing cotyledon) and to identify any abnormalities such as aberrant vessels or nods in the umbilical cord |  |  |
|  | 11 | s10q11 | Inspects the membranes for completeness |  |  |
|  | 12 | s10q12 | Notes the position of insertion of the cord |  |  |
|  | 13 | s10q13 | Inspect the cut end of the cord for the presence of two arteries and one vein |  |  |
| **Section 11. The provider adequately performs immediate postpartum care.** | 1 | s11q1 | Ensures direct strong light into the perineum |  |  |
|  | 2 | s11q2 | Ensures Mary is in a comfortable position |  |  |
|  | 3 | s11q3 | Informs and gains consent from Mary to check her perineum |  |  |
|  | 4 | s11q4 | Inspects the lower vagina and perineum for lacerations/tear and checks for increased bleeding from the cervix/cervical tear |  |  |
|  | 5 | s11q5 | Gently cleanses the vulva and perineum with clean water or a non-alcoholic antiseptic solution and dries with a clean, soft cloth |  |  |
|  | 6 | s11q6 | Makes sure that Mary is comfortable (clean, hydrated and warmly covered) |  |  |
|  | **Section 12. Documentation of AMTSL** | | | | |
|  | 1 | s12q1 | Washes hands thoroughly using soap and water and dries them/uses hand gel to clean hands |  |  |
|  | 2 | s12q2 | Records relevant details (childbirth, active management of the third stage of Labour (AMTSL), placenta examination, newborn condition) on the Mary’s record |  |  |

| **Anything from the skills drill/observations you would like to tell us?** |
| --- |
|  |
