# Supplementary material for: Midwifery care providers’ childbirth and immediate newborn care competencies: A cross-sectional study in Benin, Malawi, Tanzania and Uganda
Source: PLOS Glob Public Health. 2023 Jun 6;3(6):e0001399. doi: 10.1371/journal.pgph.0001399 (PMC10243614; doi:10.1371/journal.pgph.0001399)
Supplement: Attachment — Submitted filename: Response to Reviewers.docx [file pgph.0001399.s007.docx]

PGPH-D-22-01875

Midwifery care providers’ childbirth and immediate newborn care competencies: a cross-sectional study in Benin, Malawi, Tanzania and Uganda

Dear Reviewers and Editor,

Thank you for carefully reading the manuscript and constructive comments. We took time to review every detail and improve according to your comments. All comments have been addressed and changes to the manuscript have been saved under track changes. We are grateful to the reviewers and editor for their time and helpful comments.

| Reviewer #1 comments | Authors replies |
| --- | --- |
| Thank you for giving me the opportunity to review this paper. This is a solid study; congratulations to the authors. | Many thanks to the reviewer. |
| I think it needs better framing to situate it within the literature, particularly on methods. I am not suggesting it needs additional analysis | Thanks. We have rewritten the “Background” section to reflect this comment. |
| I also think the conceptual framing needs to be more precise to make the implications clearer. You measure knowledge and skills/behaviours (which together make up competency); you also measure perceptions of some things that will influence competencies (e.g. access to education and training resources); you also measure some other things that will influence how competencies will translate into actual practice (e.g. PPE). Likewise in the intro and discussion you talk about how the evidence base shows that pre-service education (PSE) tends to be weak; that competencies tend to be weak; how quality of care tends to be weak; and how there are many other things that influence quality of care aside from provider competency. But nowhere do you give a tight, clear explanation of how all these things fit together. Often you bounce around between these things within paragraphs. As a result, there isn’t a clear narrative running through the study. If you got your up front framing clear and precise, then you would be able to maintain this throughout the manuscript, which would improve it immensely | Many thanks for these valid points.  We have rewritten the “Background” section and in doing that we believe that we have improved the conceptual framing of the study and the points raised by the reviewer. |
| One of the things that I wasn’t entirely clear on – which will influence several comments below – is whether “success” in the skills drills was entirely down to the provider. Page 3 of S3 makes it seem like it is ensured that all of the equipment that they need is ready before they do the skills drills; in which case, any deficiencies must solely be due to the actions of the provider. However, if this is not the case, and some of the sub-optimal skills practices could be caused by facility level deficiencies (e.g., not having the requisite infrastructure and supplies), this has very different interpretations for your study. I’m assuming the former, that the skills drill is a measure of individual competency. | Thanks. For the skills drills the equipment was provided as this was a simulation and not conducted in the actual labour ward but in a separate locality within the hospitals as we did not have permission to use the labour wards. Providing same environment/equipment/tools also ensured that it was possible to compare provider competency among the countries.  Totally agree, that lack of quality of care could be caused by facility level deficiencies.  The skills drills are a measure of individual provider competency.  Now discussed in the limitation section. |
| Abstract  - Line 30: I think you need to make very explicit that when you use the word ‘behaviours’, you mean it in the context of the skill drill simulations, and not in term of actual practice. I think you also need to make explicit in the abstract that a skills drill relates to a simulation | Thanks much.  We have amended to reflect these comments so it now reflects what the assessment is based on skills drills simulations. |
| Abstract - Line 39-42. In line with my overall comment, I think you need to be clearer on what factors are other contributors to knowledge and skills aside from pre-service education; and what are the factors that may constrain competencies from translating into practice. You don’t delineate those sufficiently. You should come back and edit these lines after addressing them in the main text. | Thanks.  It reads:  *“Inadequate competency level of the midwifery care providers may be due to gaps in pre-service training but possibly related to the structural and operational facility characteristics including continuing professional development”.* |
| Line 73 typo – to what extent | Thanks – as we have updated the Background section this sentence is not any longer included. |
| - Line 68 onwards. You are (I think) essentially arguing that: 1) the competency (knowledge+skills) of the health workforce is not good enough to provide quality care (and that this is partially due to poor pre-service education), and 2) there are many other factors that affect how healthworker competency translates into quality care. But you jump between these two things without making them explicit. The flow of this para is hard to follow as a result (e.g., see the sentence that starts on line 83 – seems in a strange place). I think you need to set out a clearer framework of how everything fits together. You mention the Renfrew framework – this may be a good one (although you also mention but don’t explain Mattison on line 418). If you get this framing stronger, the narrative flow through your results and discussion will be much sharper.  - I think you need to be much more explicit on what the novel aspect of your study is, and how it contributes to the evidence base. This will be easier when your framing earlier was clearer. You make an argument that the current empirical evidence on routine care competency (as opposed to EmONC) is sparse. I would have liked to see more justification of this. I’m really surprised by the assertion that there are not more studies on the competencies of providers of routine delivery and newborn care in these countries.  - I have suggested later bringing in lines 423-434 from your discussion (on how current PSE is weak) here, as a motivator for your study. If you want to argue that you are doing the study to contribute to better PSE which will contribute to better competency which will contribute to better QoC (although there are many other factors affecting it), then this would be a coherent justification.  - I think you should set out more context from each of the four countries of study, and the evidence base (briefly) on QoC and competencies from all of them. You should also come back to this in the discussion. | Thanks. As described above we have re-worked the Background section which now reflect the points raised.  With the re-work of the Background section we have not included the sentence related to limited studies assessing competencies of providers of routine delivery and newborn care. However, we have not been able to locate many studies conducted in our study countries.  We did not conduct this study to contribute to better pre-service education. The study was conducted to inform the ALERT in-service training packages development (hopefully clear from the Background section) but we also want to highlight that skills and knowledge are insufficient - and this may stem from insufficient pre-service education.  The study highlights gaps in intrapartum (childbirth) care competencies and suppose these findings could be useful for stakeholders responsible for pre-service training in the study countries.  Related to the context of each country we have included information in the section “Study setting”. The reviewer in a comment below wrote:  *“I found lines 132 (study setting) onwards potentially misplaced. Where is this data coming from? Should this not be in the results? Why have you chosen these pieces of information? How do they relate to average places of delivery in these countries? This section overall needs more thought”.*  The information included in the “Study setting” is data collected from each of the hospitals as part of the ALERT study and not data we have collected for this study. We include this information to help setting the “scene” for the study. We have not included all details about the study countries but we are referring to the overall ALERT study protocol in the “Study setting” section if readers are interested in additional details about the ALERT study.  References:  1. Akuze J, Annerstedt KS, Benova L, Chipeta E, Dossou JP, Gross MM, et al. Action leveraging evidence to reduce perinatal mortality and morbidity (ALERT): study protocol for a stepped-wedge cluster-randomised trial in Benin, Malawi, Tanzania and Uganda. BMC Health Serv Res. 2021;21(1):1324 ).  2. Preliminary report: Dossou JP, Houngbo G, Boyi M et al. Baseline Health Facility Assessment Report ALERT. Versions 1, October, 2021.  The reviewer suggests that we focus more on QoC and competencies for all the countries. Somehow we think this is covered in the frameworks discussed in the Background section. |
| Study Setting  - I found lines 132 onwards potentially misplaced. Where is this data coming from? Should this not be in the results? Why have you chosen these pieces of information? How do they relate to average places of delivery in these countries? This section overall needs more thought | Thanks. Please refer to our reply above. |
| Study Tools  - It would be nice to reflect on these tools; how similar tools have been used in other settings/are they novel etc. What are the strengths and limitations of using these kinds of tools? See a recent study in India that used similar approaches - Where there is no nurse: an observational study of large-scale mentoring of auxiliary nurses to improve quality of care during childbirth at primary health centres in India - PMC (nih.gov) – and the references cited within (40 onwards). You should try to situate your study within the methodological literature. | This is a very valid point and many thanks for highlighting. We have added some reflections to the section where we discuss study limitations. Reviewer #2 also asked about the study tools – please see our reply below to reviewer #2.  We read the BMJ Open paper with great interest.  The authors of the paper refer to different tool e.g. Structured Clinical Examinations (OSCEs) used to develop their study but does not share their final study tool as well as the “checklist” they used. It reads: *“….Tool development was facilitated by CARE programme developers, India-based obstetric experts and international experts in BEmONC training. The same tool was used in all assessments. A panel of three nurses from the USA and India (Delhi and Bihar) assessed the face and content validity (by indicating if a particular item should be included or not) of the tool”.* The authors do not provide any information regarding which elements were changed in the tool based on the validation. |
| Pre-Test  - There are various typos here: proof read carefully. | Thanks, we have carefully checked for typos. |
| Knowledge assessment results p. 15  - Lines 265- are about background characteristics; I would make this a separate section. | Thanks, we have amended accordingly. |
| Line 283 onwards – this is where I think your lack of precision in the intro doesn’t help the clarity here – you mention lots of different things that impact different parts of your study/implicit framework, so what you are actually showing here isn’t clear. You should split this into more discrete sections, and refer back to an overall framework. Many of these facts are included in Figure 3 which isn’t referenced here. Some of these overlap with the Study setting section. | Thanks. We have now amended and have split into two sections - Participant characteristics and knowledge assessment results. |
| Line 285 is about written protocols. What is the logical implication of this? Do we assume? that not having a written protocol will undermine skills/behaviour? Or knowledge? Or both? | Thanks.  Practical protocols/clinical guidelines are fundamental for ensuring quality health care. These are often underutilized and/or poorly implemented, inefficient planned or even outdated.  Lack of practical protocols/clinical guidelines imply that providers have to rely on their own knowledge and initiative to develop skills and behaviours and are left to informal  decision making. Providers need to access, be supported and trained in ensuring they can adhere to practical protocols/clinical guidelines to provide quality of care.  The Background section now covers this element. |
| Line 289 is actually about knowledge. But what is the right answer? Is there an objective right answer? Later on you talk about different countries having different policies – how does this affect here? | Thanks. Knowing when to measure fetal heart rate monitoring is about knowledge and essential in providing childbirth care. Consulting different guidelines have indicated that recommendations vary. Suppose the right answer will depend on which guideline is implemented.  The point here is that some of the study countries do not have protocols including guidance on fetal heart rate monitoring. The results showed that study participants provided different answers to the questions related to the frequency of fetal heart rate monitoring which may indicate lack of knowledge and/or needed guidance. |
| Line 302. Is there a right answer? Or does it depend on country policies. How do we interpret this? | Thanks. Evidence suggests that companionship throughout labour and birth is very beneficial for a positive childbirth experience and reduce mistreatment of women during childbirth.  It is a strong recommendation in the WHO intrapartum guideline (<https://www.who.int/publications/i/item/9789241550215>).  Sure, we understand the concern. If companionship is not a policy, the providers will not know. However, our aim was to illustrate knowledge and skills against internationally accepted recommendations. |
| Some of these (e.g. use of partograph line 304) are about clinical practice not knowledge. Even accompaniment is about what actually goes on, not necessarily knowledge. (I hope this gives a concrete example of why you need a clearer framework and more precision). Likewise line 318 on reporting – this is about actual practice not knowledge. | Thanks. While the implementation is about practice, we were looking here at theoretical knowledge. As we describe now in the introduction, we appreciate the importance of the working environment, but in this study, we wanted to understand better the theoretical knowledge and skills. We hope the new introduction situates the study clearer. |
| Lines 325 onwards are definitely not about knowledge! | Thanks. The study tools were developed at the start of the COVID-19 pandemic and we were interested in getting some more information about provider competencies related to childbirth and COVID-19. At that time the topic related to COVID-19 was very political sensitive in the study countries and these questions were the only which got approved for inclusion. Agree with the reviewer that this is not specific to knowledge but at least the providers need to know if PPE is available and the importance of changing between patients, and aware of any in-service training provided. We also think this was a good opportunity to show that despite such a huge public health problem, there was little training, guidance and resources provided to help providers deal with it/provide quality care.  We would appreciate guidance from the editor related to the comment. |
| Skills drills results  - Line 346-349 on overall averages – good to include these in Figure 4, not just the supplemental file. | Figure 4 includes all the averages or did we misunderstand the reviewers comment?  Please note now figure 3 after revision. |
| Discussion  - I think you should try to make the connection between knowledge and skills – to what extent are some of the weak simulated skills and behaviours due to knowledge defects? Can you draw threads through the two sets of data?  - Overall, this would all be much sharper if you had more precision up front. For example, Line 377: presumably supervision, education and training resources, and managerial support are things that impact retention of knowledge and skills/new learning – so are reasons why competencies are partial; it’s not just about poor PSE. Yet Line 378 you start talking about PPE, which won’t affect any of the things you have measured – and will be one of the things that affect the transmission between competencies and practice (which you don’t measure), which you need to reflect on. | Thanks. We have now re-worked the Discussion.  Insufficient knowledge and skills may also limit the providers ability to shape their working environment. |
| Line 380 – what does the fact that there are different guidelines mean for the interpretation of this study? | Thanks. Please refer to our reply above regarding fetal heart rate monitoring. |
| Line 390 – the evidence base should also have been referred to in the introduction (see my comment there). Some of them (e.g. lines 401-405), it’s not clear whether they are measuring competencies or actual practice; the text needs more precision | Thanks. This refers to the actual practice during the skills drill’s simulation. To perform a task here “*rapid and initial newborn assessment*” as a provider your practice is based on your skills and behaviours. |
| Again you mix literature findings on i) low competencies, ii) weak PSE, iii) other factors that constrain the translation of competencies into practice – without weaving these into a narrative story about what your findings tell us. | Thanks. We have now incorporated these comments. |
| - The Mattison framework is not explained (line 418). Again you bounce between this point (factors undermining providers) to the next sentence (line 420) about lack of investment in education. The narrative flow needs to be stronger. | Thanks. As we re-worked the Background section the Mattison framework is included and we have worked on the narrative flow. |
| Line 423-434 – this point about low quality of education – you should bring this into the introduction as a motivator of your study. You do not assess the quality of education in your study, so it does not fit as well in your discussion (but would be important to bring into your introduction). | Thanks. We have now included this in the Background section. |
| - Come back to the country level variations; some comment here. Are these in line with outcomes in these countries (representativeness of your sample issues aside). | Thanks. The findings in our study are in line with some key health outcomes such as for example the maternal mortality ratio and neonatal mortality rate. We have added text to address this comment in the discussion. |
| Strengths and limitations  - You should reflect more on the limitations of only measuring simulated skills and behaviours, and not also actual practice, and the evidence base on these types of methods (See earlier comment). | Thanks. We have amended the limitations section to include these points. |
| Reviewer #2 | Authors replies |
| Thank you for undertaking this complex research work. The result of the study is a good starting point to evaluate the competencies of midwives regarding labor and childbirth. Although this study is already informative in itself, the study will motivate more research work around the evaluation of the competencies of midwives in providing labor and childbirth beyond EMONC | Many thanks for taking time to review our manuscript and providing comprehensive feedback.  We certainly also hope that the study will prompt some additional research to ensure better health outcomes for women and newborns. |
| Title  The title is passive | The authors are not sure about the comment that “the title is passive”. We have kept the original title but are open for suggestions. |
| Abstract  How did you sample the participants?  What analyses did you perform? | We have added details to the abstract. |
| Background:  The study rationale, significance, and purpose are clearly stated.  Are there any previous studies documenting the competencies of care providers with respect to childbirth and immediate newborn care? | Many thanks.  Limited studies are available for sub-Saharan Africa and our study countries. Identified studies focus mainly on basic and comprehensive emergency obstetric and not physiological childbirth and immediate newborn which was our focus.  An example of a similar study even though different methods were applied as the study used interviewer administered questionnaire and observation checklist. “Knowledge and practice of immediate newborn care among midwives and nurses in public health facilities of Afar regional state, Northeast Ethiopia” (<https://bmcpregnancychildbirth.biomedcentral.com/articles/10.1186/s12884-019-2581-3>).  As reviewer 1 highlighted (please see above) a similar study has also been conducted in India. |
| Methods  How did you recruit the study participants?  What sampling techniques did you use to sample your participants?  Did you test for the validity of the tools that you developed? If so, how did they perform?  How long did it take to administer the questionnaires? | Recruitment: all midwifery care providers including doctors providing midwifery care in the 16 ALERT project hospital maternity units were invited to take part in the knowledge assessment – please see under the “*Target population and sampling” section* where the sampling techniques are described.  Sampling techniques: for the self-administered questionnaire all providers in the project hospitals were invited to take part.  Approximately one third of the midwifery care providers who took part in the knowledge assessment were randomly selected and invited to take part in the skills and behaviour simulation assessment.  It took approximately 30 minutes for the participants to fill in the questionnaire.  Tool validation: many thanks for stressing this point.  We assessed face and content validity. The assessments were done by ALERT team members who did not take part in the design of the tools. We have added details on the study tool section in the “Study tools” section.  The tools were also pre-tested please see details in section “Pre-test” section. |
| Results  The results are robust and highly informative. Thank you | Thank you very much. |
| Discussion  Most of the glaring study limitations are well highlighted, thank you.  If your findings are not generalizable, can they be transferrable given that your study is purely quantitative? | Thanks. We have now reworded and it reads:  Due to the diversity of settings, it may not be possible to generalize the study’s findings but likely that similar deficiencies would also be observed in midwifery care providers in other similar settings. |
