# Supplementary material for: Midwifery care providers’ childbirth and immediate newborn care competencies: A cross-sectional study in Benin, Malawi, Tanzania and Uganda
Source: PLOS Glob Public Health. 2023 Jun 6;3(6):e0001399. doi: 10.1371/journal.pgph.0001399 (PMC10243614; doi:10.1371/journal.pgph.0001399)
Supplement: Attachment — Submitted filename: Response to Reviewers.docx [file pgph.0001399.s008.docx]

PGPH-D-22-01875

Midwifery care providers’ childbirth and immediate newborn care competencies: a cross-sectional study in Benin, Malawi, Tanzania and Uganda

Dear Reviewers and Editor,

Thank you for carefully reading the revised manuscript and constructive comments. We took time to review the details and improve according to your comments. All comments have been addressed and changes to the manuscript have been saved under track changes. We are grateful to the reviewers and editor for their time and helpful comments.

Please note that we now have included reference to the latest available maternal mortality estimates published 23 February 2023 (<https://www.who.int/publications/i/item/9789240068759>). Which replaces the previously used reference: WHO, UNICEF, UNFPA, The World Bank Group, United Nations Population Division. Trends in maternal mortality: 2000 to 2017: estimates by WHO, UNICEF, UNFPA, The World Bank Group and the United Nations Population Division. Geneva: World Health Organization, 2019.

| Reviewer #1 comments | Authors replies |
| --- | --- |
| This article is looking very strong, and the previous comments have been incorporated well. Many congratulations to the reviewers. | Many thanks. Great to know that the reviewer considers our manuscript improved and strong. We certainly appreciate these details and additional comments. |
| A couple of full stops are missing after the edits (lines 33, 71, 208, 435). 465 has one that is not needed. Just do a final proofing. | Thanks. We have made these edits. |
| Lines 298-308 – which are about working environment – should come in the section starting line 311 (titled knowledge and working environment assessment results) rather than the participant characteristic sections (particularly as the data is reported in figure 1 and not table 2). | We have amended accordingly. |
| Line 315 (and around): I still feel you could help a non-medical reader like myself by saying “the majority responded correctly that…”. Not saying what is correct or not correct makes it hard for the reader to interpret the information. In the discussion section, you are explicit about this (you use ‘correct’ on line 399) but it would be good to also have it here. If there are areas where what is correct is not obvious but the issue is inconsistency, then I would make that explicit (in line with your response to my earlier comment). | We have included the recommended changes and hopefully now clear for the reader. |
| Line 347-348 – this writing is confusing, just have another look at the sentence | Thanks. We have reworded to ensure a better flow. |
| Is line 427 (participants further…) about your study or about the Ethiopia study introduced on line 421? Because it is not a new paragraph, it is not clear. I assuming it is about your study and the start of a new point – in which case, bring it to a new para and signpost it accordingly. I.e. make it clear that your study also identified some of the working environment challenges that midwives faced which will compromise QoC. I still think you could be more precise that these are both factors that will prevent midwives from increasing their knowledge (access to training resources, supervision) and factors that will prevent them from translating their knowledge into QOC (e.g., lack of equipment and supplies). | Thanks for highlighting this. We have rephased. |
| The COVID-19 text (431-435) – it feels a bit strange to introduce it in the discussion, because you did not describe it in the findings (apart from some of the tables). I think you should either mention it also in the findings section and keep it here, or remove it from here. | This is mentioned in our results section. Please see lines 365-372 in the in the manuscript with track changes and manuscript (clean) lines 350-357. |
| 474 limitations not limitation. In this section, did you want to reference back to the challenge of not including DODs to assess practice? | Many thanks for noting this. We have amended accordingly. |
| Line 506 and 507 (we should do this) seem to clash with 501-502 (we should do more research to work out what to do) – just think about rewording this slightly? | Thanks. We reworded. |
| Reviewer #2 comments | Authors replies |
| I thank the authors for addressing the previous comments. | Many thanks and we appreciate that the reviewer finds the revisions acceptable. |
| However, a second look into the methods indicate that the study group is so diverse (doctors, midwives, nurses, nurse-midwife). The WHO/FIGO/ICM came up with the terminology 'skilled health personnel' to describe this diverse groups.  I suggest the authors adopt this terminology for this paper. | Many thanks for this observation and comment.  The joint statement by WHO, ICM and FIGO regarding the critical role of the skilled attendant was published in 2004 (<https://apps.who.int/iris/handle/10665/42955>).  It reads: *“an accredited health professional — such as a midwife, doctor or nurse — who has been educated and trained to proficiency in the skills needed to manage normal (uncomplicated) pregnancies, childbirth and the immediate postnatal period, and in the identification, management and referral of complications in women and newborns”.* This statement is policy document and has not been accompanied by a measurement framework but has often been used to inform measurement approaches.  A major measurement limitation is the varying interpretations of the 2004 definition of skilled health personnel. Underpinning this variation is the lack of guidelines, lack of standardization of job titles and functions for skilled health personnel, as well as gap between international standards and actual provider competencies in many settings.  To address these challenges, a Task Force, composed of inter-agency groups, which included the WHO, UNICEF, UNFPA, the International Confederation of Midwives (ICM), the International Council of Nurses (ICN), the International Federation of Gynecology and Obstetrics (FIGO) and the International Pediatric Association (IPA), initiated a process to clarify and refine the definition of the widely used term “skilled birth attendant” (SBA) with the aim of ultimately informing improved and operationalized measurement of births attended by “skilled health personnel”.  A revised definition was published in 2018 *(*[*https://apps.who.int/iris/handle/10665/272817*](https://apps.who.int/iris/handle/10665/272817)*).*  The 2018 Definition reads:  *Skilled health personnel, as referenced by SDG indicator 3.1.2, are competent maternal and newborn health (MNH) professionals educated, trained and regulated to national and international standards.*  *They are competent to:*  *(i) provide and promote evidence-based, human-rights- based, quality, socio-cultural sensitive and dignified care to women and newborns;*  *(ii) facilitate physiological processes during labour and delivery to ensure a clean and positive childbirth experience; and*  *(iii) identify and manage or refer women and/or newborns with complications. In addition, as part of an integrated team of MNH professionals (including midwives, nurses, obstetricians, paediatricians and anaesthetists), they perform all signal functions of emergency maternal and newborn care to optimize the health and well-being of women and newborns.*  *Within an enabling environment, midwives trained to International Confederation of Midwives (ICM) standards can provide nearly all of the essential care needed for women and newborns (In different countries, these competencies are held by professionals with varying occupational titles).*  A scoping review identified a total of 102 unique cadres names from 36 LMIC countries of providers providing childbirth care (https://journals.plos.org/plosone/article?id=10.1371/journal.pone.0211576).  Currently a project is ongoing to develop a measurement framework for the 2018 Definition of skilled health personnel.    With this background we prefer to keep the terminology used in our manuscript which is also in line with the study protocol. |
